# Time varying dynamics of hallucinations in clinical and non-clinical voice-hearers

**DOI:** 10.1016/j.nicl.2023.103351

**Published:** 2023-02-14

**Authors:** Theresa M. Marschall, Sanne Koops, Sanne G. Brederoo, Joana Cabral, Branislava Ćurčić-Blake, Iris E.C. Sommer

**Affiliations:** a University of Groningen, Department of Psychiatry, University Medical Center Groningen, Groningen, The Netherlands; b Centre for Eudaimonia and Human Flourishing, Linacre College, University of Oxford, Oxford, UK; c Life and Health Sciences Research Institute, School of Medicine, University of Minho, Braga, Portugal

**Keywords:** Auditory verbal hallucinations, Psychosis, Schizophrenia, Functional magnetic resonance imaging, Dynamic connectivity, Continuum hypothesis of psychosis

## Abstract

Auditory verbal hallucinations (AVH) are frequently associated with psychotic disorders, yet also occur in non-clinical voice-hearers. AVH in this group are similar to those within clinical voice-hearers in terms of several phenomenological aspects, but non-clinical voice-hearers report to have more control over their AVH and attribute less emotional valence to them. These dissimilarities may stem from differences on the neurobiological level, as it is still under debate whether the mechanisms involved in AVH are the same in clinical and non-clinical voice-hearers. In this study, 21 clinical and 21 non-clinical voice-hearers indicated the onset and offsets of AVH during an fMRI scan. Using a method called leading eigenvector dynamics analysis (LEiDA), we examined time-varying dynamics of functional connectivity involved in AVH with a sub-second temporal resolution. We assessed differences between groups, and between hallucination and rest periods in dwell time, switching frequency, probability of occurrence, and transition probabilities of nine recurrent states of functional connectivity with a permutation ANOVA. Deviations in dwell times, switching frequencies, and switch probabilities in the hallucination period indicated more erratic dynamics during this condition regardless of their clinical status. Post-hoc analyses of the dwell times exhibited the most distinct differences between the rest and hallucination condition for the non-clinical sample, suggesting stronger differences between the two conditions in this group. Overall, these findings suggest that the neurobiological mechanisms involved in AVH are similar in clinical and non-clinical individuals.

## Highlights

• Dynamic functional connectivity was assessed on a sub-second level.
• Hallucination periods showed more erratic switching behavior than rest periods.
• Most characteristics did not differ significantly between clinical and non-clinical voice hearers.
• Differences in dwell time suggest dysfunction of the pallidum in the clinical group.

## 1 Introduction

Auditory verbal hallucinations (AVH) are often seen as a hallmark of psychosis and occur in approximately 60–80 % of schizophrenia patients (Larøi et al., 2012, Slade and Bentall, 1988, Waters et al., 2014). However, they can present as a symptom of various psychotic and neurological disorders and are also reported in otherwise healthy individuals (Eversfield and Orton, 2019, Inzelberg et al., 1998, Larøi et al., 2012, Toh et al., 2015). People in the latter group are commonly referred to as non-clinical or healthy voice-hearers, as they experience frequent and complex AVH similar to those in psychotic disorders, yet they neither show a need for care nor meet the criteria to be diagnosed with any disorder besides experiencing AVH (Baumeister et al., 2017). Whereas the lifetime prevalence for voice-hearing is assumed to lie around 10 % in the general population (Beavan et al., 2011), the regular experience of complex AVH, such as full sentences, as seen in non-clinical individuals appears closer to 1 % to 2 % (Johns et al., 2002, Kråkvik et al., 2015). AVH in non-clinical individuals are a relevant phenomenon to investigate, as they allow us to study the experience of AVH unaffected by antipsychotic medication or other symptoms commonly seen in patients with psychosis. However, it is yet to be determined whether AVH in clinical and non-clinical groups arise from the same underlying mechanisms.

Studies that directly compared AVH in clinical and non-clinical individuals identified a number of similarities in subjective features of the perceived voices. They found that audiological features of the AVH, such as descriptions of the loudness, number of voices, and relative location of the voice(s) perceived by non-clinical voice-hearers resemble those of AVH in people with psychosis (Baumeister et al., 2017, Daalman et al., 2016, Daalman et al., 2011a, Sommer et al., 2010). Both groups also indicate that their AVH are indistinguishable from real voices (Moritz and Larøi, 2008). In contrast, there is a large discrepancy between AVH in psychosis and in non-clinical voice-hearers with regards to the emotional appraisal and valence of the voices heard. Specifically, multiple studies show that psychotic patients experience more distress from their AVH and report more negative content (Cottam et al., 2011, Daalman et al., 2011b, Daalman et al., 2011a, de Boer et al., 2021, Honig et al., 1998, Sommer et al., 2010). A recent study from our group showed that the emotional valence of hallucinated words and sentences is more negative in patients versus non-clinical voice hearers (Corona-Hernández et al., 2022). In addition, clinical voice-hearers seem to endure longer and more frequent AVH than non-clinical voice-hearers (Andrew et al., 2008, Daalman et al., 2011a, Diederen et al., 2010, Honig et al., 1998, Slotema et al., 2012).

Several models have been proposed to explain the differences and similarities between clinical and non-clinical voice-hearers, with the most prominent hypothesis being that the experience of hallucinations in both groups lay on a continuum of increasing distress or need for care (Baumeister et al., 2017, Claridge, 1994, Linscott and van Os, 2013, Linscott and van Os, 2010, Toh et al., 2022, van Os et al., 2009). According to this theory, the experience of AVH in the clinical population is not necessarily due to their psychosis, but rather comes from the same or a similar mechanism as AVH in non-clinical individuals. It is assumed that the negative appraisal and life impact of the AVH or the actual risk for developing psychosis for these individuals is assumed to lay on a continuum (Claridge, 1994, Johns et al., 2014, Toh et al., 2022, Waters and Fernyhough, 2019). This means that phenomenologically similar AVH can occur in the presence or absence of a diagnosis within the psychosis spectrum, but the negative valence and impact on people seems to be related to a higher disease severity. While this model has been criticized to be an oversimplified view on psychosis (David, 2010, Kaymaz and van Os, 2010, Linscott and van Os, 2010), it offers an explanation for the similarities between clinical and non-clinical voices in assuming overlap between the underlying mechanisms. Simultaneously, it may suggest the involvement of two separate working mechanisms in the occurrence of AVH, with one causing the perception of voices in both clinical and non-clinical voice-hearers, whereas the other is responsible for the level of emotional valence.

Studies investigating the neurobiological underpinnings of AVH provide further evidence for differential working mechanisms of clinical and non-clinical AVH. For example, differences have been reported with regard to effortful attention (van Lutterveld et al., 2010), white matter integrity (de Weijer et al., 2013), brain activity during a verbal fluency task (Diederen et al., 2010), and cortical thickness (van Lutterveld et al., 2014). Interestingly, non-clinical voice-hearers seem to resemble healthy individuals more than clinical voice-hearers in terms of dopamine synthesis capacity (Howes et al., 2013b, Howes et al., 2013a). Yet, when directly comparing the blood-oxygenation-level-dependent (BOLD) signals during the occurrence of AVH in the same sample that will be assessed in the current study, the two groups could not be distinguished (Diederen et al., 2012). Instead, several brain areas were identified with significant overlap in their activation. In line with this, connectivity patterns of non-clinical voice-hearers during AVH are more alike those of clinical voice-hearers than of non-voice-hearing control participants (Diederen et al., 2013). Taken together, direct comparisons of neurobiological mechanisms between clinical and non-clinical voice-hearers show varying results.

As hallucinations are a rather dynamic experience (McCarthy-Jones et al., 2014a, McCarthy-Jones et al., 2014b, Nayani and David, 1996), with changes in content and emotional appraisal over time, it may be difficult to disentangle the mechanisms involved when only considering static methods, that is, averaging the brain activity involved in AVH over the timeframe of a functional scan (Allen et al., 2014, Hutchison et al., 2013). In recent years it has been shown that functional connectivity does not remain stagnant, but rather constitutes transitions between several discrete connectivity states (Hutchison et al., 2013, Lurie et al., 2020). Using this approach to functional connectivity may shed new light on the neurobiological underpinnings of AVH. Specifically, investigating higher order statistics describing brain dynamics and the interaction of the brain networks involved may allow detection of more subtle changes in the brain activity underlying AVH in different populations. Previously, such a dynamic approach to functional connectivity has been useful in the detection of biomarkers in a variety of mental and neurodegenerative disorders, such as depression, schizophrenia, bipolar disorder, Alzheimer’s disease, and Parkinson’s disease (Demirtaş et al., 2016, Díez-Cirarda et al., 2018, Farinha et al., 2022, Gu et al., 2020, Nguyen et al., 2017, Rabany et al., 2019). Additionally, it enables the detection of intra- and inter-individual differences in a variety of cognitive domains (Hutchison et al., 2013, Shine et al., 2016). Regarding AVH in schizophrenia, recent studies on dynamic functional connectivity patterns showed a decrease in dwell time in networks characterized by the default mode network (DMN) anticorrelated with either task active networks (Weber et al., 2020) or language related networks (Geng et al., 2020). In other words, participants who experienced AVH had shorter periods of the DMN detaching from networks related to tasks or language compared to those without AVH.

The aim of this study is to describe the brain activity of clinical and non-clinical voice-hearers by investigating the characteristics of their dynamic functional connectivity during the occurrence of AVH as compared to rest. For this, participants’ individual AVH time courses were assessed during functional magnetic resonance imaging (fMRI), using a balloon press paradigm. This can be seen as an advantage over trait studies investigating AVH. Whereas trait studies assess the overall brain activity during a scan as related to the general tendency of experiencing hallucinations (Zmigrod et al., 2016), a button press paradigm provides the means to directly measure the activity related to the hallucination period. By asking the participants to indicate the onsets and offsets of their hallucinations, we can assure that the brain activity we investigated is directly associated with the experience of AVH in our sample. Since AVH occur spontaneously and do not follow predefined onsets and offsets, leading eigenvector dynamics analysis (LEiDA) can be suitable to identify changes close to the individual AVH time course and detect specific patterns related to their occurrence. Using this data-driven approach we expect to find dynamic functional connectivity patterns directly related to AVH, which should differ from the behavior of the networks during the resting periods. Additionally, we expect more subtle differences between the clinical and non-clinical voice-hearers, related to the emotional valence attributed to the AVH. Therefore, this study furthers research on AVH by taking a dynamic approach, which may reveal previously undetected differences between clinical and non-clinical voice-hearers. The aim of this study is twofold. First, we want to assess differences between clinical and non-clinical voice hearers on a dynamic level. Second, the hallucinatory and non-hallucinatory state within the patients will be compared to identify changes in the dynamic characteristics related to the presence of AVH.

## 2 Methods

### 2.1 Participants

A total of 42 participants who experience AVH on a regular basis were included in this study. 21 participants were clinical voice-hearers, i.e., voice-hearers diagnosed with a psychotic disorder (10 diagnosed with schizophrenia, 2 with schizoaffective disorder, and 9 with psychosis Not Otherwise Specified (NOS)). The other 21 were considered non-clinical voice-hearers, as they perceive voices, but did not meet the criteria to be diagnosed with a psychiatric disorder other than anxiety or depressive disorder in full remission according to Diagnostic and Statistical Manual of Mental Disorders, Fourth Edition, (DSM-IV, “American Psychiatric Association Diagnostic and Statistical Manual of Mental Disorders (DSM-IV),” 2012). This was assessed by an independent psychiatrist using the Comprehensive Assessment of Symptoms and History (CASH, Andreasen et al., 1992) and Structured Clinical Interview for personality Disorder (SCID-II, First et al., 2011). Here, we will shortly describe the sample and recruitment; for a more detailed overview of the recruitment, inclusion criteria, and exclusion criteria see Diederen et al. (2012).

Non-clinical voice-hearers were part of a larger study on hallucination proneness in the general Dutch population described in Sommer et al. (2010). Individuals who scored high on items 8 and 12 of the Launay Slade Hallucinations Scale (Larøi et al., 2004) (‘In the past, I have had the experience of hearing a person’s voice and then found that no-one was there’; ‘I have been troubled by hearing voices in my head’), were invited to the University Medical Center Utrecht to undergo further psychiatric assessment. From this group, 21 individuals were selected for this study, as they experienced sufficient AVH during the MRI scan for analysis. Non-clinical voice-hearers were not diagnosed as psychosis NOS, due to them not showing any social or professional dysfunction, while also not being perturbed by their AVH.

Clinical voice-hearers were selected from another study by our group described in Slotema et al. (2011). These 21 individuals were selected to match to the non-clinical voice-hearers, Matching variables were used in the following order: sex, handedness, age, years of education, total duration of the hallucinations during the scans, number of AVH during the scans, and average duration of a AVH during the scans. The groups matched adequately on most but not all variables (see Table 1).

**Table 1:** Demographic data and AVH characteristics.

		Non-clinical voice-hearers (n = 21)		Clinical voice-hearers (n = 21)		Statistic	p-value
Demographics							
Age	Mean (SD)	46.524 (11.622)		39.952 (11.320)		t(40) = 1.856	0.071
Sex	female; n (%)	4 (19.048 %)		7 (33.333 %)		χ2(1) = 0.493	0.483
Handedness	right; n (%)	14 (66.667 %)		13 (61.905 %)		χ2(1) = 0	1
Years of education	Median (IQR)	14 (1)		13 (4)		D = 0.381	0.095

**AVH characteristics^a^**			Description		Description		

AVH frequency^b^	Median (IQR)	4 (1)	>1/day	6 (1)	Almost continuous	D = 0.619	0.001
AVH duration^c^	Median (IQR)	2 (0)	Few minutes	2 (2)	Few minutes	D = 0.333	0.194
AVH location^c^	Median (IQR)	2 (3)	Inside head and near ears	1 (1)	Inside head	D = 0.286	0.358
AVH loudness^c^	Median (IQR)	2 (0)	Little softer than own voice	2 (2)	Little softer than own voice	D = 0.190	0.841
AVH explanation origin^c^	Median (IQR)	3 (1)	>50 % external	2 (2)	<50 % external	D = 0.429	0.042
AVH emotional valence^c^	Median (IQR)	0 (1)	Almost no negative content	3 (2)	<50 % negative content	D = 0.714	<0.001
AVH total distress^c^	Median (IQR)	0 (1)	Almost no discomfort and disruption of life	6 (1)	Severe distress and disruption of life	D = 0.952	<0.001
AVH controllability^c^	Median (IQR)	1 (1)	Most of the time	3 (3)	Sporadically	D = 0.524	0.006
AVH N different voices	Median (IQR)	7 (7)		4 (18)		D = 0.333	0.194
AVH age onset	Median (IQR)	7 (13)		21.31 (12)		D = 0.476	0.017

**AVH during scan^d^**							

total AVH duration	Median (IQR)	107.715 (104.201)		158.402 (157.647)		D = 0.286	0.365
Average AVH duration	Median (IQR)	8.684 (5.511)		10.161 (9.856)		D = 0.286	0.365
Number of AVH	Median (IQR)	15 (9)		13 (21)		D = 0.190	0.841
Percentage of scan time	Median (IQR)	0.224 (0.217)		0.33 (0.328)		D = 0.286	0.365

Note: This table provides general characteristics of the sample, a description of the AVH characteristics in the past 3 months as assessed with an adapted version of the Psychotic Symptom Rating Scales (PSYRATS; Haddock et al., 1999), and information about the AVH that were measured during the scan.

a: AVH characteristics in the past 3 months were assessed using the Psychotic Symptom Rating Scales (PSYRATS; Haddock et al., 1999).

b: possible range: from 0 to 6.

c: possible range: from 0 to 4.

d: AVH during scan is based on the onsets and offsets the participants indicated during scan time.

All participants were screened for drug use prior to participation, by testing urine samples for opiates, amphetamines/XTC, cocaine, and cannabis.

Both studies were approved by the Humans Ethics Committee of the University Medical Center Utrecht, The Netherlands. All participants provided written informed consent.

### 2.2 Data acquisition

During the acquisition of the fMRI scans, participants were instructed to keep their eyes closed and indicate the on- and off-sets of their AVH with balloon-squeezes.

For each patient, fMRI images (n = 800) were recorded using a 3D-PRESTO pulse sequence with paralleled imaging (SENSE) in two directions with the following parameters: TR = 21.75 ms, TE = 32.4 ms, 64 mm × 64 mm × 40 mm acquisition matrix, field of view (FOV) = 224 mm × 160 mm, voxel size = 4 mm3, flip angle 10°, number of slices (coronal) = 40. The scan sequence combined a 3D-PRESTO pulse sequence with parallel imaging (SENSE) in two directions using a commercial 8-channel SENSE head-coil and resulted in full brain coverage in 609 ms.

To improve the realignment and coregistration steps of the preprocessing, an additional 40 identical scans with a flip angle of 27° (fa27) were acquired, as PRESTO images may possess limited anatomical contrast.

Subsequently, high resolution structural T1-weighted images were collected for anatomical reference (TR = 9.86 ms, TE = 4.6 ms, FOV = 240 mm × 160 mm × 168.00 mm, flip angle 8°, voxel size = 0.875 mm × 0.875 mm × 1 mm).

All structural and functional images were acquired using a Philips Achieva 3.0 Tesla scanner (Philips Medical Systems, Best, The Netherlands) equipped with a commercial 8-channel SENSE head coil.

### 2.3 Data preprocessing

Preprocessing of the fMRI data was performed in SPM 12 as well as in-house scripts in MATLAB and included the following steps: (1) within-subject realignment using the mean fa27 as reference, (2) coregistration of the mean fa27 and T1 weighted anatomical scan, (3) segmentation of the anatomical scan, (4) spatial normalization to Montreal Neurological Institute (MNI) space using the transformation between the fMRI data and a structural T1 scan of the same participant and a transformation between the same structural T1 scan and the MNI template, which included resampling to 2 × 2 × 2 mm voxel size, and (5) smoothing of the functional scans with an 8 mm full-width at half maximum (FWHM) Gaussian kernel to reduce spatial noise.

Next, the functional data were filtered with a band-pass temporal filter (0.04 – 0.07 Hz) using a fifth-order Butterworth filter (Glerean et al., 2012). Finally, the average time series of 90 cortical and subcortical regions of the Automated Anatomical Labeling (AAL) atlas (Tzourio-Mazoyer et al., 2002) were extracted.

### 2.4 Dynamic functional connectivity with LEiDA

**Fig. 1:**
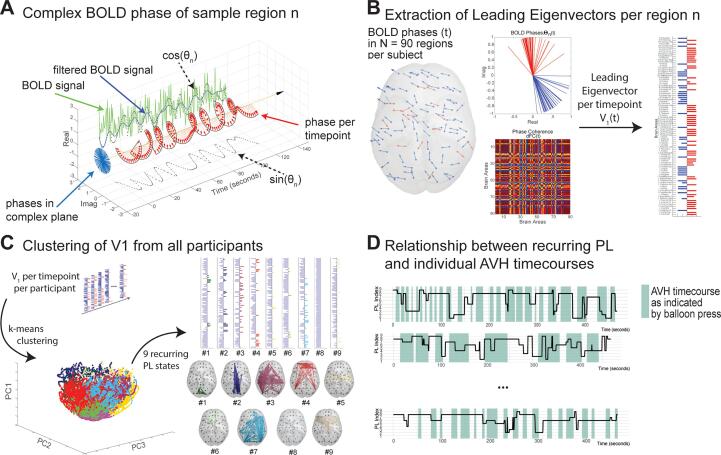
Graphical overview of the methods. A) Depiction of the Hilbert transform performed on the mean BOLD signal for each of the N = 90 regions of the AAL atlas. The BOLD signal is plotted in green, the filtered BOLD signal in blue and the sine and cosine of the signal in dotted lines. Red arrows depict the phase at each timepoint. B) An example of the phase of the BOLD signal and the leading eigenvector at a given timepoint t. The matrix shows the phase coherence between each of the N = 90 areas at timepoint t. Phases are also depicted in the cortex and the complex plane. Phases marked red have a different sign than the majority of areas indicating a separate network. The leading eigenvector is extracted from the phase coherence matrix for each timepoint for each subject. C) k-means clustering was performed on all leading eigenvectors and 9 recurring phase locking patterns (PL) were identified. D) The characteristics of the PL transitions were related to the AVH time courses indicated by the participants. Shaded areas indicate AVHs. (For interpretation of the references to color in this figure legend, the reader is referred to the web version of this article.)

The dynamic functional connectivity was assessed using LEiDA, a method that allowed us to determine changes in connectivity on a quasi-instantaneous level, by utilizing phase coherence between brain areas (Cabral et al., 2017). An overview of the steps of this method can be seen in Fig. 1.

First, a Hilbert transform was performed to estimate the instantaneous phase of the fMRI signals for each of the 90 brain regions as defined by the AAL atlas per time point. The Hilbert transform imparts a phase shift of 90° on the signal, representing it on the complex plane. To prevent edge effects commonly caused by the Hilbert transform, 20 volumes were discarded (the first 10 and final 10 volumes of the scan), resulting in 780 timepoints for analysis. Phase coherence between each pair of brain regions for a given time point was calculated as the cosine between the differences of their phases using the following formula:

$$dFC\left(n,p,t\right)=\cos\left(\theta \left(n,t\right)-\theta \left(p,t\right)\right),$$

with θ(n,t) being the phase of the BOLD signal in region n at a given time t, and θ(p,t) being the phase of the BOLD signal in region p at a given time t. This resulted in a symmetric coherence matrix consisting of the undirected phase coherence of the size 90 × 90 × 780 (90 brain regions and 780 time points) for each subject, indicating the similarity between the BOLD signal of two regions n and p at time t. Phase coherence of 1 indicates temporal coherence, that is, strong similarity between the phases, whereas a phase coherence of 0 suggests orthogonal signals.

Next, the leading eigenvectors of each coherence matrix were extracted to reduce dimensionality of the data per time point, leaving us with a 1 × 90 vector per time point, or 780 × 90 matrix per subject. The 42 individual matrices were concatenated to a 32,760 × 90 matrix containing all leading eigenvectors from all subjects and k-means clustering was performed with K ranging from 3 to 15 clusters to determine recurrent functional states or phase locking patterns (PL) across the entire sample. The Dunn index (Dunn†, 1974) was calculated to determine the ideal number of clusters K. PL are reoccurring network configurations that may reflect known resting-state network or parts thereof (Alonso Martínez et al., 2020, Vohryzek et al., 2020). Yet they may also be specific to the sample or pathology at hand. Similarities between the identified PL and known networks described by Yeo et al. (2011) were assessed using correlation coefficients.

### 2.5 Description of functional connectivity characteristics

Four types of characteristics describing the behavior of the PL were extracted per condition for each of the participants. To determine whether a time point was considered rest or hallucination, a 1 by 780 array was created based on the individual’s balloon presses during the scan. Each element of this array was either assigned a 1 for AVH or a 0 for rest. As scanning was performed at a time resolution with the TR of 0.6 s, AVH time courses needed to be rounded to adhere to the scanning time. Several rounding methods were tested, and rounding to the nearest integer resulted in the lowest deviation from the original duration of AVH. The differences between the rounded and original AVH time courses can be found in the Appendix.

Differences between groups (clinical vs non-clinical voice-hearers) and condition (hallucination vs rest) were assessed for:

1. *Switching frequency*: The number of transitions between functional states per second
2. *Probability of occurrence*: The percentage of time points during which a certain cluster occurred.
3. *Mean dwell time*: The average length of a cluster being consecutively active
4. *Switch probability:* The likelihood of a given state transitioning into any of the other states

**Fig. 2:**
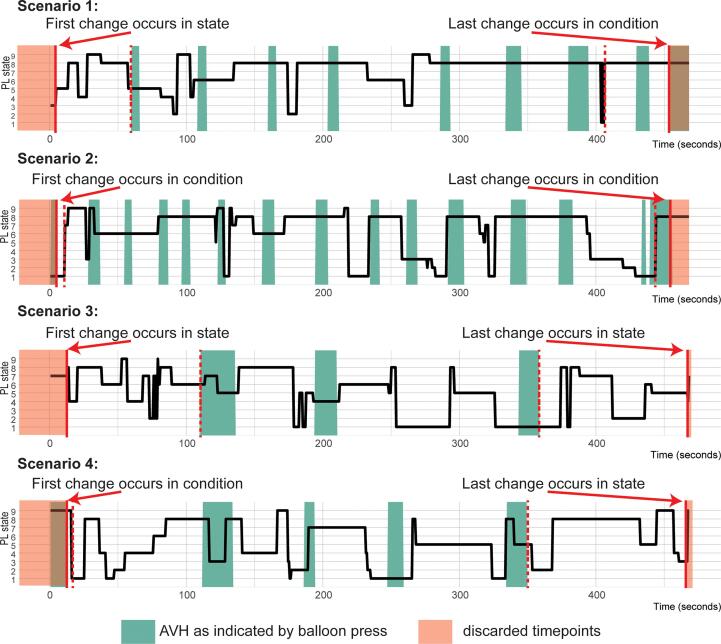
Illustration of the selection of the individual time range from which the state characteristics were extracted. Each graph shows an example of the transitions between the different PL states (indexed on the y axes) over time (shown on × axis), as well as the individual AVH time courses. Green patches indicate active AVH. Red lines show the chosen starting/ending point used for analysis, whereas dashed red lines show the other non-chosen possibility. Red background marks the timepoints discarded. For example, in scenario 1 the first change occurs in a state transition (marked red), the first condition transition happens much later (red dashed line), therefore we chose to use the state transition as onset and discarded timepoints prior to this (red background). (For interpretation of the references to color in this figure legend, the reader is referred to the web version of this article.)

For every participant these state characteristics were extracted for the two conditions (rest and AVH). As the first and last state of the overall scanning time had no definitive start or end point, we excluded them from the analysis. Per participant the individual starting point was selected by the first change either in condition (Fig. 2, scenario 2) or state (Fig. 2, scenario 1). The end point was chosen based on the last change respectively.

For the extraction of the mean dwell time and the analysis of the switching probability of one condition, we set the time points of the other condition to 0. This means that during the AVH condition each active time point would be assigned a PL state whereas all time points of the rest condition, i.e., the time points between two consecutive AVH occurrences would be set to 0 and vice versa. This way we avoided inflated dwell times in case two consecutive events within a condition would concatenate into one prolonged occurrence of the same state. Aside from that, this approach made it possible to include this “0″ condition into the analysis of the switching probability. This way we were able to identify whether certain PLs tend to precede or follow one of the two conditions more often.

### 2.6 Statistical analyses

#### 2.6.1 Demographics

All statistical analyses were performed using R Statistical Software (R Core Team, 2021). For the comparison of the two groups with respect to their demographics, t-tests were used for normally distributed continuous variables and chi-square tests for categorical variables. For all other variables describing the two groups, namely years of education, variables describing the AVH characteristics in the past 3 months (see Table 1), and variables describing the AVH during scan (see Table 1), we performed an independent samples Kolmogorov-Smirnov tests to examine whether the variables showed similar distributions. Missing data on the age of the AVH onset for 3 non-clinical and 5 clinical voice hearers was imputed per group using mean imputation.

#### 2.6.2 State characteristics

Differences in the state characteristics were assessed with permutation repeated measures ANOVA implemented in the aovperm function of the R package permuco (Frossard and Renaud, 2021). Each test was performed with 10,000 permutations. Condition, group, and PL state, as well as the interactions between these variables were included into the model as independent variables. The index of the PL state to which the switch happened was also included as an independent variable in the analysis of the switch probability. Significant interactions between PL state occurrence and any combination of the other variables were of interest as they indicated an effect of the variable on the characteristics of specific PL states. Measures which were extracted more than once per participant, for example, separate measures for each condition or PL state, were handled as within-subject factors. The analysis of the switching probability included the aforementioned “0″ condition to identify specific transition patterns related to the onset or offset of a condition. This means that the time points that do not belong to the condition of interest would be set to 0, therefore transitions in and out of this state indicate changes in condition. For significant interactions of the ANOVA analyzing the switch probability, post hoc permutation t-tests were performed on all possible interactions within a certain PL state using the function pairwise.perm.t.test of the R package RVAideMemoire (Hervé, 2022) with 10,000 permutations. Post hoc tests were corrected using false discovery rate (FDR) for the number of tests.

#### 2.6.3 Shift in time course

Aside from our main analyses, we also assessed whether the delay in the BOLD response may affect our results, as the BOLD signal is known to peak 4–6 s after being presented with a stimulus (Bénar et al., 2002, Kobayashi et al., 2006, Krakow et al., 2001, Raichle and Mintun, 2006). This issue was addressed by shifting the individual AVH time course by 8 TR, that is 4.8 s, and repeating the analyses. This value was chosen as it best approaches the center of the 4–6 s delay. Discords between these two approaches should be interpreted with caution.

## 3 Results

### 3.1 Demographics

The statistical comparison between clinical and non-clinical participant data is reported in Table 1. The two groups did not differ significantly regarding their sex (χ2(1) = 0.49267; p = 0.483), handedness (χ2(1) = 0; p = 1), age (p = 0.095), and years of scholar education (p = 0.071).

In terms of AVH characteristics, the groups were matched at p > 0.05 with regard to their AVH duration, location, loudness, as well as the number of different voices they experience. A difference was present in terms of age onset, explanation origin, and controllability of the AVH, although this difference was not sufficient to be statistically significant when correcting for family-wise error rate FWER (p > 0.05/17 = 0.0029, the Bonferroni-corrected threshold considering that 17 independent comparisons were made). Considering the FWER, significant differences between clinical and non-clinical voice hearers were only detected in terms of AVH frequency, emotional valence, and total distress (p ≤ 0.001).

The groups were matched at p > 0.05 regarding the AVH during the MRI scan in terms of total duration, average duration, number of events, and percentage of scan time.

### 3.2 Phase locking patterns

**Fig. 3:**
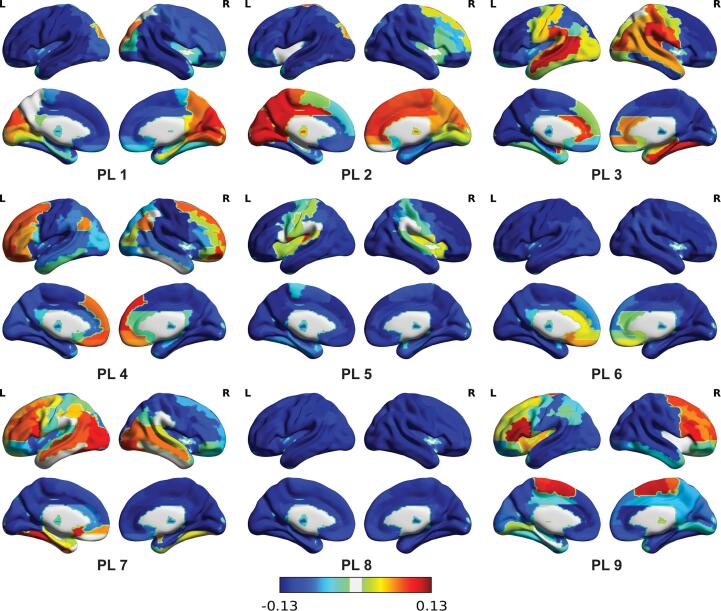
Overview of the 9 phase locking patterns (PL) used for analysis. The 32,760 eigenvectors detected across all participants were clustered using k means into K = 9 clusters, each represented by it’s cluster centroid. The cluster centroids are 1x90 vectors corresponding to the recurrent PL patterns, where the 90 elements represent the relative phase of the 90 brain regions of the AAL atlas. Here the relative phase is used to scale the color of each brain region projected on an inflated cortex.

**Fig. 4:**
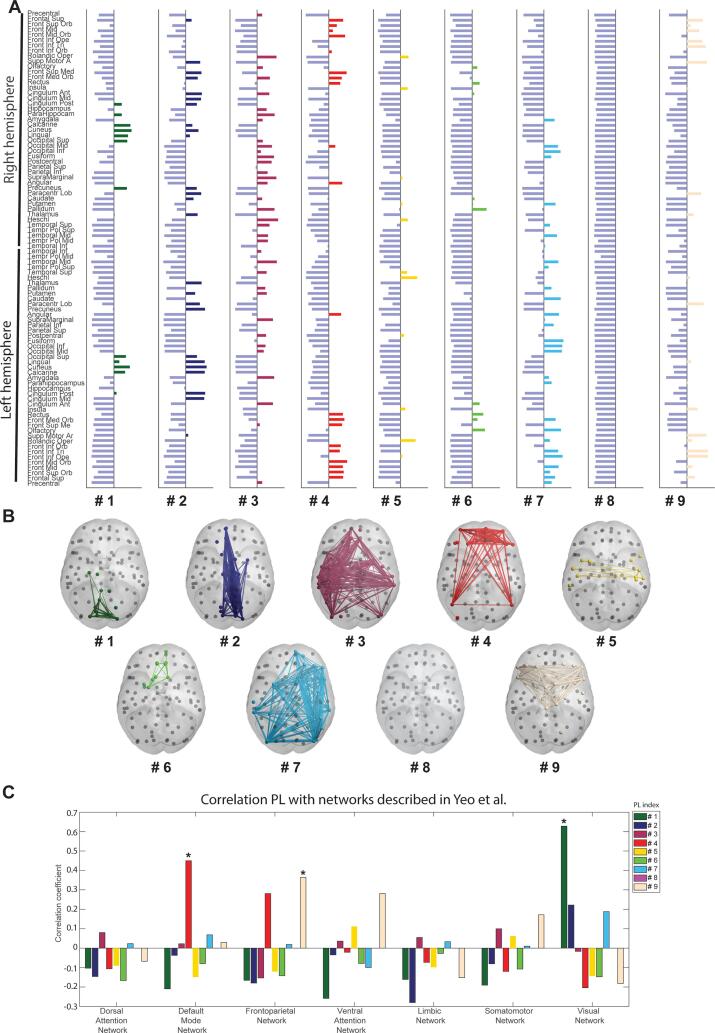
Description of the Phase Locking patterns obtained with K = 9 and their overlap with resting-state networks A) Leading eigenvectors of each of the PL per ROI of the AAL atlas. The horizontal bars show the values of the cluster centroid, Vc, for each of the k = 9 PL states identified. Each horizontal bar represents the phase of the BOLD signal per ROI of the AAL atlas. B) Graphical representation of the PLs in cortical space. Each sphere represents one of the ROIs of the AAL atlas. Colored spheres indicate the nodes of the PL. Edges are shown if their value is higher than the mean; A version of this graph without edges can be found in the appendix. C) Correlation coefficients with known networks described by Yeo et al., 2011. Significant correlations are marked with an asterisk. Positive correlations indicate a spatial overlap or similarities between the PL and network. Negative correlations occur when there is little to no overlap. A graphical representation of the Yeo et al networks can be found in the appendix.

The optimal partition of PL patterns into clusters was obtained for K = 9, according to the Dunn index (maximum of 0.0058 for K = 9, see Appendix). These PL patterns are depicted in Fig. 3 and an ordered overview of the eigenvector elements per region for each PL can be found in the appendix. Most of the PLs were not significantly related to the networks described by Yeo et al. (2011); see Fig. 4). Negative correlations between these networks and the PL indicate little to no spatial overlap. PL 1 showed a significant positive relationship with the visual network (c = 0.628, p < 0.001), PL 4 with the default mode network (c = 0.450, p <.001), and PL 9 with the frontoparietal network (c = 0.364, p <.001) after correcting for multiple comparisons.

While the other correlations between the PL the networks described by Yeo et al. (2011) were not significant, there are some similarities between the visual network and PL 2 and PL 7, the frontoparietal network and PL 4, the ventral attention network and PL 9, and the somatomotor network and PL 9 (see Fig. 4). PL 3 is mainly located around the bilateral temporal cortex and also includes parts of the limbic system, such as the amygdala and hippocampus. PL 5 includes the bilateral Heschl’s gyri and Rolandic Operculum, suggesting it to be an auditory network. With regions such as the Pallidum, the rectal gyrus, and the olfactory gyrus, PL 6 is focused around the inferior frontal gyrus. In line with other studies employing the LEiDA method, PL 8 is a state of global phase coherence. This state has been assumed to reflect the global signal (Bennett et al., 2016, Cabral et al., 2017, Keller et al., 2013).

For a detailed overview of the regions included in each PL we refer to the appendix.

#### 3.2.1 Switching frequency

The analysis of the switching frequency showed a significant difference between the two conditions, AVH and rest (F(1,40) = 11.189, p =.002). Switches occurred more frequently during the AVH periods (mean = 0.110) than during rest (mean = 0.085). Clinical and non-clinical voice-hearers did not differ from each other in terms of switching frequency (F(1,40) = 0.032, p =.860). The interaction between the two variables also showed no significant difference (F(1,40) = 1.873, p =.183). Graphs depicting these distributions can be found in the Appendix (A9–A10).

#### 3.2.2 Probability of occurrence

Neither the groups (F(1,40) = 0.065, p =.802) nor conditions (F(1,40) = 0.134, p =.999) differed significantly on probability of occurrence of PL. Probability of occurrence showed a significant main effect on PL state (F(8,320) = 68.597, p <.001), indicating that certain PLs were more likely to occur than others. PL 8 had the highest probability of occurrence with a mean of 45.37 % and PL 7 the lowest with a mean of 4.39 % followed by PL 3 with a mean of 4.40 %. None of the interactions were significant (group*condition: F(1,40) = 0.032, p =.992; group* PL: F(8,320) = 1.608, p =.124; condition* PL: F(8,320) = 0.971, p =.459; condition*group* PL: F(8,320) = 0.560, p =.812). For graphs depicting these distributions we refer to the Appendix (A11–A12).

#### 3.2.3 Mean dwell time

**Fig. 5:**
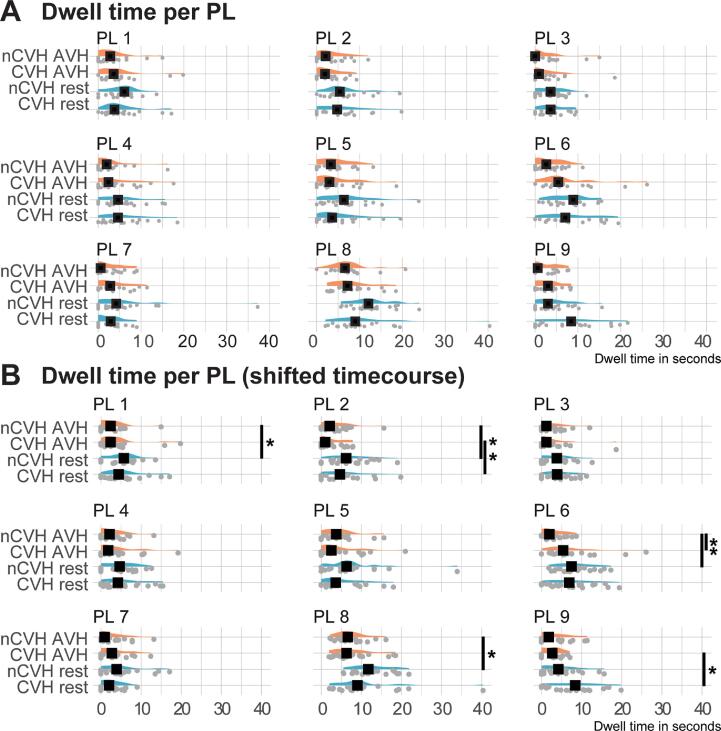
Distribution underlying the mean dwell times without and with the shift in time course. A) Interaction effects of group, condition, and PL state in on mean dwell time. The y-axes show the interaction between conditions and group (AVH in orange, rest in blue; nCVH = non-clinical voice hearers, CVH = clinical voice hearers). The x-axis depicts the dwell time in seconds. Grey dots are the individual data points. Black boxes indicate the median of the distribution. No differences were significant. B) Interaction effects of group, condition, and PL state in on mean dwell time following the time shift. The y-axes show the interaction between conditions and group (AVH in orange, rest in blue; nCVH = non-clinical voice hearers, CVH = clinical voice hearers). The x-axis depicts the dwell time in seconds. Grey dots are the individual data points. Black boxes indicate the median of the distribution. Significant differences are marked with asterisks. (For interpretation of the references to color in this figure legend, the reader is referred to the web version of this article.)

The mean dwell time differed significantly between the two conditions (F(1,40) = 23.326, p <.001). The consecutive time spent in the same PL was longer during rest (mean = 6.313) than during AVH (mean = 3.999). In addition, PL state significantly affected mean dwell time (F(8,320) = 14.736, p <.001). PL 8 had the highest dwell time with a mean of 9.655 and PL 3 the lowest with a mean of 3.283. There was no main effect of group (F(1,40) = 0.160, p =.699). None of the interaction effects were significant (group*condition: F(1,40) = 1.588, p =.208; group*PL: F(8,320) = 1.586, p =.125; condition*PL: F(8,320) = 1.556, p =.135; condition*group*PL: F(8,320) = 1.681, p =.101; see Fig. 5A). Graphs depicting these distributions can be found in the Appendix (A13–A14).

#### 3.2.4 Switch probability

In terms of the probability of transitioning from one state to another, there were no significant main effects of group (F(1,40) = 0.298, p =.499), condition (F(1,40) = 0.251, p =.603), switching from a particular PL state (F(9,360) = 0.830, p =.470), or switching to a particular PL state (F(9,360) = 0.125, p =.985). Regarding the two-way interactions, there was only one significant interaction between the PL the switch occurred from and the PL the switch occurred to (PL_from*PL_to: F(71,3240) = 10.073, p <.001), indicating that there are certain transition patterns that were more likely than others. The other two-way interactions were not significant (group*condition: F(1,40) = 0.713, p =.386; group*PL_from: F(9,360) = 0.523, p =.733; group*PL_to: F(9,360) = 0.251, p =.375; condition*PL_from: F(9,360) = 1.033, p =.375; condition*PL_to: F(9,360) = 0.673, p =.646). As for interactions including three variables, we found an effect of *condition*, *PL from*, and *PL to* (F(71,3240) = 1.674, p <.001), indicating that certain switches were more common in one condition over the other. The interactions including the variable group were not significant (group*condition*PL_from: F(9,360) = 1.770, p =.114; group*condition*PL_to: F(9,360) = 0.695, p =.624; group*PL_to*PL_from: F(71,3240) = 0.686, p =.983). Similarly, the interaction between all four variables did not show a significant effect(F(71,3240) = 1.159, p =.169).

#### 3.2.5 Switch probability – Post hoc comparisons:

To further inspect the significant three-way interaction, post hoc comparisons were conducted by comparing all possible pairs of the remaining variables (*condition, PL from, and PL to).* All tests were corrected for multiple comparisons using FDR*.* Here we present the results focusing on the effects of condition. Tables including all comparisons can be found in the appendix.

**Fig. 6:**
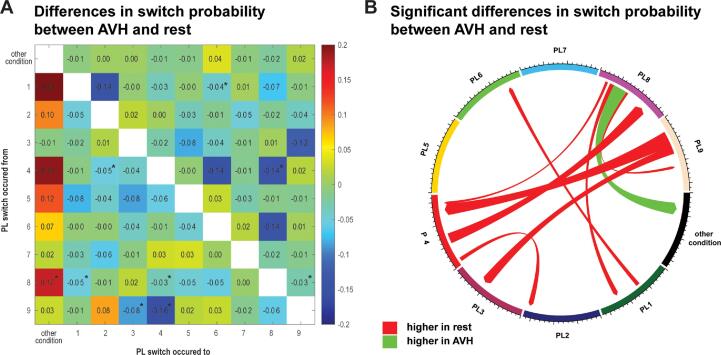
Results of the post hoc comparison of the switch probabilities. The left side of the figure presents difference in switch probabilities, with the significant ones marked with and asterisk. The right side shows a chord diagram of the significant differences, red lines indicate negative values green lines positive values. Here mean switch probabilities of the rest condition were subtracted from the mean switch probabilities of the AVH condition, i.e., negative values indicate higher mean values during rest. (For interpretation of the references to color in this figure legend, the reader is referred to the web version of this article.)

We found nine pairs of PL states whose switching patterns differed significantly between the hallucination and the rest periods in our sample. The majority of these differences were related to an increased switch probability to or from PL 8, PL 4, and PL9 (see Fig. 6).

#### 3.2.6 Shift in time course

The majority of the results obtained after shifting the time courses to account for the delayed hemodynamic response overlapped with the outcome from the main analysis and can be found in the appendix. However, differences were found in the mean dwell time and the switch probability.

As for the mean dwell time, shifting the time course resulted in a significant interaction between the three variables condition, group, and PL (F(8,320) = 2.193, p =.025). Further post hoc comparisons revealed four instances where there was a difference between the AVH and rest periods in the non-clinical group. PL 1, PL 2, PL 6, and PL 8 of the non-clinical group showed a significantly lower mean dwell time during the AVH periods than during rest. The clinical group had a significantly lower mean dwell time of PL 2 and PL 9 during their AVH periods compared to the rest periods. Additionally, we found a higher mean dwell time of PL 6 for the clinical group during hallucinations as compared to the non-clinical group. A graphical overview of these results can be seen in Fig. 5.

Regarding the switch probability, shifting the time course resulted in an interaction effect of the variables group condition and PL the switch occurred from (F(9,360) = 3.414, p =.010), which was not present in the initial analysis. However further post hoc comparisons of this interaction did not survive correction for multiple comparisons using the FDR method. Additionally, the interaction between condition, PL the switch occurs towards, and the PL the switch occurs from, which was significant in the initial analysis, does not reach significance following the shift in the AVH time course (F(71,3240) = 1.228, p =.096).

## 4 Discussion

This study set out to investigate whether characteristics of dynamic functional connectivity differ between clinical and non-clinical voice-hearers and are specifically tied to the occurrence of AVH (i.e., state) or can better be considered a trait of voice-hearers. This comparison provides insights into the neurobiological processes occurring during the experience of AVH. Nine phase locking patterns (PLs) were identified underlying the brain activity during an fMRI paradigm in which participants indicated the onsets and offsets of their AVH. The dwell time, switching frequency, probability of occurrence, and switch probability of these PLs were assessed.

No distinct differences between clinical and non-clinical voice-hearers were found with respect to the switching probability, probability of occurrence, and switching frequency of the PLs observed, suggesting that these characteristics are similar in clinical and non-clinical participants. Our sample also showed no significant differences in their dwell time of the identified PLs in our main analysis, again supporting the idea of similar brain activity underlying the AVH in both groups. Only after shifting the AVH time course, the dwell time differed significantly on group level, with clinical voice-hearers spending on average more consecutive moments in PL 6 during their AVH compared to non-clinical voice hearers. One of the main regions of this PL, namely the pallidum, has previously been associated with hallucinatory behavior in clinical individuals; dopamine transporter availability in this area was positively related to the occurrence of AVH in schizophrenia patients (Artiges et al., 2017). Importantly, the clinical participants used antipsychotic medication, which may alter metabolism in this area. Additionally, increased grey matter volume of areas similar to the PL has been described in schizophrenia patients with AVH (Sampedro et al., 2021). Changes in the pallidum were seen in patients with treatment-resistant AVH compared to patients without AVH. Therefore, increased prolonged utilization of this PL may contribute to the presence of AVH. Alternatively, it may be a consequence of their antipsychotic medication use.

However, the general patterns of our results suggests that both groups exhibit similar brain activity on the dynamic scale, which has also been suggested in earlier studies investigating the mechanisms underlying AVH in the same sample of clinical and non-clinical voice-hearers when comparing their brain activity with more static techniques (Diederen et al., 2013, 2012). Especially the results of Diederen et al. (2012) directly localizing areas of overlapping neuronal activity during AVH in a clinical and non-clinical population, support a common mechanism in the two groups. Similar mechanisms underlying the AVH in these groups would be consistent with the idea of a continuum of hallucination proneness (Baumeister et al., 2017, Linscott and van Os, 2013, Linscott and van Os, 2010, Toh et al., 2022, van Os et al., 2009).

Importantly, based on our analyses we cannot make inferences about the similarity (i.e., absence of a difference) between the groups, due to the frequentist nature of the statistical tests used. Therefore, it may also be possible that the heterogeneity of the individual neurobiological processes of the AVH, for example due to differences in AVH content, may not show on a group level. Indeed, AVH are found to exhibit a large variability in features within and between populations (Larøi et al., 2012, Waters and Fernyhough, 2017, Woods et al., 2015). Further characterization of certain subtypes based on phenomenological and linguistic characteristics of the voices experienced may help identify more similar clusters of individuals experiencing AVH regardless of the initial diagnosis (Chang et al., 2015, McCarthy-Jones et al., 2014a, McCarthy-Jones et al., 2014b, McCarthy-Jones et al., 2014a, McCarthy-Jones et al., 2014b, Stephane et al., 2003). This approach could homogenize the effects of the voices experienced by the sample. While a recent study assessing the linguistic and phenomenological features of clinical and non-clinical voice-hearers revealed that the AVH in these individuals could be separated into two distinct clusters, it is worthwhile to mention that these clusters did not necessarily resemble the two main participant groups, but rather demonstrated that both clinical and non-clinical individuals were present in each of the clusters (Corona-Hernández et al., 2022). Consequently, differences in the underlying mechanisms of the AVH may be better reflected by a more phenomenology-based approach instead of clinical status.

Deviations between the two conditions, hallucination and rest, were more pronounced than differences between the two groups. With higher switch frequency in the hallucination condition and higher mean dwell times during rest, our findings suggest a more erratic network behavior during hallucinations. This was further supported by the post-hoc comparisons with the clinical as well as the non-clinical group exhibiting decreased dwell times during AVH as compared to the resting periods. Prior studies have shown that the networks in AVH behave differently from those in non-hallucinating individuals. Shortened microstate durations have frequently been reported in EEG studies examining the temporal dynamics of AVH (Nishida et al., 2013, Rieger et al., 2016, Strelets et al., 2003). It has been proposed that these deviations in temporal characteristics cause disruption of the network, which in turn emphasizes aberrant information processing and source misattribution (Honcamp et al., 2022, Kapur, 2003, Lehmann et al., 2005). These are two mechanisms often associated with the occurrence of AVH (Aleman et al., 2003, Allen et al., 2005, Corlett et al., 2019, Johns et al., 2014, Pinheiro et al., 2019).

Similar results were reported in an fMRI study by Weber et al. (2020), in which a significant decline in dwell time was found in patients with schizophrenia and hallucinations as compared to patients with schizophrenia without hallucinations, with regard to a task positive network that opposed the default mode network. Interestingly, this finding was not repeated when comparing the dwell time of the same network between schizophrenia patients and healthy controls. In line with this, Geng et al. (2020) identified a network with significantly shorter dwell times in schizophrenia patients with AVH than in schizophrenia patients without AVH. This network was characterized by an anti-correlation between the default mode network and the language network. Symptoms of non-clinical voice-hearers are often considered to be part of a continuum between the general population and schizophrenia patients. As part of our population does not meet the criteria to be diagnosed with a psychotic disorder, finding similar patterns as reported by Weber et al., 2020, Geng et al., 2020 may point towards a possible neural substrate involving more erratic switching behavior in AVH compared to rest regardless of a diagnosis.

Our post hoc analysis of the switch probability revealed that the majority of the differences can be attributed to a lower likelihood to switch between certain PL during AVH compared to rest. While this initially seems counterintuitive considering the decreased dwell time in AVH, we expect this also reflects a more erratic behavior of the brain. Instead of following the more consistent switching patterns seen during the rest period, the brain appears to follow a less distinct path. Generally, aberrant switching patterns have been suggested to affect the interplay between the networks involved and may increase the likelihood of cognitive disfunction (Honcamp et al., 2022).

In our study, switches between PL 8, PL 4, and PL 9 are mainly affected. Similar to other studies using the LEiDA method, we found a state of global phase coherence in PL 8. While the exact function of this type of state is still unclear, it has been suggested to represent the global signal commonly found in fMRI studies (Bennett et al., 2016, Cabral et al., 2017, Keller et al., 2013). Changes in the temporal characteristics of this state have been related to a variety of function and disfunction, including cognitive performance (Cabral et al., 2017), depressive symptoms (Alonso Martínez et al., 2020, Figueroa et al., 2019), insight (Larabi et al., 2020), and schizophrenia (Farinha et al., 2022). It is likely that the occurrence of this state of global coherence facilitates the integration or separation of different brain areas and could therefore contribute to cognitive function (Kringelbach et al., 2015, Nomi et al., 2017). Deviation found in the temporal characteristics of this network may therefore point towards more general disfunction related to AVH.

With PL 9 being related to the frontoparietal and showing similarities with the ventral attention network (VAN), it is likely that abnormalities in the utilization of this network are directly related to the occurrence of hallucinations. As the VAN contains regions of the right frontoparietal cortex known to be related to the perception of AVH, such as Broca’s area/inferior frontal gyrus (Ćurčić-Blake et al., 2017, Plaze et al., 2011, Sommer et al., 2008), a lower number of switches out of this network may contribute to maintenance of the AVH. Interestingly, the supplementary motor area (SMA) also strongly contributes to this PL. The SMA plays an important role in error monitoring, therefore deviations in the utilization of this network may hamper the ability to disentangle AVH from reality. Several studies have reported evidence for involvement of the SMA in the occurrence of AVH (Alderson-Day et al., 2015, Alonso-Solís et al., 2015, Ćurčić-Blake et al., 2017, Lavigne et al., 2015, Mechelli et al., 2007). Alternatively, SMA involvement may be due to the balloon pressing paradigm (Hanakawa et al., 2008).

In a similar way the occurrence of AVH may be associated with PL 4, a state that mainly contains areas of the ventral medial prefrontal cortex (VMPFC) and is positively correlated to the default mode network (DMN). Deviations in the DMN have frequently been reported in the context of AVH. It is assumed that due to its role in self-referential processes it may be directly involved in the occurrence of AVH (Alderson-Day et al., 2016, Alderson-Day et al., 2015, Bastos-Leite et al., 2015, Ćurčić-Blake et al., 2017, Northoff, 2014, Northoff and Qin, 2011). Our findings show a change in the utilization of the PL4 when comparing the two conditions, suggesting a decrease in switches to the DMN during AVH. In line with our findings, it is generally assumed that abnormal interactions between the DMN and other resting state networks are related to AVH (Alderson-Day et al., 2016, Alonso-Solís et al., 2015, Jardri et al., 2013). Deviations in the utilization of the DMN are assumed to affect the monitoring of internally generated sounds, causing them to be misinterpreted as external sounds, i.e., resulting in AVH (Frith, 2005, Wible et al., 2008).

In a similar vein, Garrison et al. (2019) suggested that the VMPFC contributes to the ability to distinguish whether the perceived voices stem from inside or outside sources. Evidence for this idea has been provided by Konu et al. (2020), who demonstrated the involvement of the VMPFC in the formation of intrusive thought. Interestingly, several studies have demonstrated the VMPFC to be specifically involved in the onset of AVH (de Pierrefeu et al., 2018, Fovet et al., 2022, Hugdahl et al., 2022, Lefebvre et al., 2016, Shergill et al., 2004). Hugdahl et al. (2022) as well as Shergill et al. (2004) demonstrated changes in VMPFC activity preceding AVH. Activity in this area has also been successfully used to distinguish between AVH and non-AVH periods (de Pierrefeu et al., 2018, Lefebvre et al., 2016), forming further support for its involvement in the presence of AVH. Considering that only our analysis without the 4.8 *sec* delay contained deviations in the switch probability, it is likely that the aberrant temporal characteristics observed in this study are in line with earlier findings regarding the role of the VMPFC in the onset of AVH.

It has been suggested that rest periods within a task paradigm do not fully reflect baseline activity of the brain (Fair et al., 2007, Stark and Squire, 2001). It is, therefore, possible that brain activity does not fully return to its baseline, especially in individuals who experience rapid consecutive occurrences of AVH. Thus, comparing rest and hallucination periods does not necessarily encompass very distinct mechanisms. Considering that our sample showed larger differences in dwell time between AVH and rest periods in the non-clinical sample, it may be possible that the return to baseline activity is accelerated in this group compared to clinical voice-hearers. Several studies have shown that the role of brain activity during rest periods may not be neutral and an increase of other processes, such as self-referential or introspective processes including mind-wandering (Andrews-Hanna, 2012, Binder et al., 2009, Binder et al., 1999). Therefore, having a better distinction between rest and AVH periods in the non-clinical group raises the possibility that self-referential thoughts are more intact in this sample. This finding may point towards a way to differentiate the mechanisms involved between the two groups as deviations in self-reference have been suggested to contribute to AVH in schizophrenia (Allen et al., 2007, Bentall and Slade, 1985, McGuire et al., 1993).

The shift in time course has resulted in significant differences in mean dwell times across conditions and groups in several PL that were not present in the initial analysis. We assume that this may be due to switches occurring around the onset or offset of the AVH. Therefore, the shift in time course could have caused transitions between certain PL states moving from one condition into the other. The temporal dynamics in the period preceding a task or perception have been shown to be related to the subsequent performance (Ekman et al., 2012, Sadaghiani et al., 2015, Weisz et al., 2014), suggesting active changes occurring at these moments. Differences in dwell time have been reported more frequently when comparing tasks to rest conditions with temporal dynamics stabilizing more during tasks as compared to rest periods (Chen et al., 2015, Elton and Gao, 2015, Liu and Duyn, 2013). This has been argued to be related to the cognitive demands of the task (Chen et al., 2015, Gonzalez-Castillo and Bandettini, 2018). The decrease in dwell time during AVH as seen in our results may therefore be related to the tendency of AVH to occur in situations with decreased focused attention (Krans et al., 2015, Nayani and David, 1996). As our results correspond to reports of shorter microstates during AVH found in EEG studies (Nishida et al., 2013, Rieger et al., 2016, Strelets et al., 2003, and the general consensus of a delay in BOLD response (Bénar et al., 2002, Kobayashi et al., 2006, Krakow et al., 2001, Raichle and Mintun, 2006), we assume that the finding following the shift may be more sensible. Yet, the differences in the results suggest that our finding on dwell time may be less robust than the other findings and should be handled with caution.

### 4.1 Limitations

The interpretation of the results of this study is subjected to several limitations. First, relying on the participants to indicate the onsets and offsets of their AVH using button presses during the fMRI scan may not fully reflect the time course of their AVH, as participants might have different reaction times (Jain et al., 2015) and different capacity in reflecting on their inner experience (Allen et al., 2007, Bentall and Slade, 1985, McGuire et al., 1993). This may have especially influenced the results extracted from the relatively short periods of AVH, as well as the transitions between AVH and rest.

In line with this, it should also be pointed out that the temporal resolution of fMRI studies is suboptimal for studying the individual time courses of AVH. As the resolution of the data acquisition during fMRI studies is limited by the TR of the scan, exact mapping of the AVH time course is not possible. In this study, the PRESTO sequence with an exceptionally low TR (0.6 s) was used to minimize this risk. We have attempted to mitigate part of this limitation by using several rounding methods in our time course, to reduce the loss of useful time points. A second issue regarding the time resolution of our study is due to the nature of fMRI measurements. As fMRI indirectly measures brain activity by assessing the change in BOLD effects the peak in the signal is usually delayed by 4–6 s (Bénar et al., 2002, Kobayashi et al., 2006, Krakow et al., 2001, Raichle and Mintun, 2006). This complicates precise estimates of the onsets and offsets of the AVH. However, the combination of a delayed reaction of the participant and the delayed BOLD response may have at least partly canceled each other out. We decided to provide the results both with and without a shift in the AVH time course, so that potential effects of the BOLD delay can be taken into account.

The use of temporal filters during preprocessing may have affected the reliability of our results. While this step has been commonly implemented in other studied using the LEiDA method (Alonso Martínez et al., 2020, Capouskova et al., 2022, Figueroa et al., 2019, Larabi et al., 2020), recent work has shown increased within-subject reliability when assessing the whole-frequency spectrum (Vohryzek et al., 2020). This may have resulted in a limited overlap between the PL states identified and known networks.

Last, the heterogeneity of the AVH experienced by an individual (McCarthy-Jones et al., 2014a, McCarthy-Jones et al., 2014b, Nayani and David, 1996), for example due to differences in duration, intensity, or content, may have affected the power of our analyses. It is possible that these individual differences are reflected in the dynamic characteristics of brain activity underlying AVH and caused an increase in the variance in our data and, therefore, diminished power to detect differences on a group level (Lipsey and Hurley, 2009). For future studies either a more individualized approach or a larger sample size may be beneficial.

### 4.2 Future directions

While this study explored the difference between the underlying mechanisms of AVH in clinical and non-clinical individuals, it may be of interest to also assess how these groups compare to a control group or a comparable group of patients with psychosis but without AVH. If symptoms of the clinical and non-clinical individuals indeed lay on a continuum, aberrant brain activity may not be detectable by only comparing the two samples presented in this study. On the one hand, adding a neurotypical control group could help quantify the deviations from baseline activity and test whether the brain activity of the non-clinical individuals lay between the control and clinical populations. On the other hand, a psychosis group without hallucinations would be useful to identify psychosis specific mechanisms that are not associated with AVH. While these individuals may not be able to hallucinate during the scan, a listening or imaginary task could be used to imitate the attentional aspects of our paradigm. This would provide some additional insight into the process of returning to rest periods.

## 5 Conclusion

This study aimed to quantify the dynamic brain activity underlying AVH in clinical and non-clinical voice-hearers. The use of the LEiDA method granted a large advantage over other dynamic functional connectivity measures, as it allowed us to study the dynamics of brain activity on a sub second time scale (Cabral et al., 2017, Figueroa et al., 2019). By doing so, we provided a fine-grained insight into the dynamics involved in the brain activity during both AVH experience and rest in our sample. The button press paradigm used in this study permitted us to examine the individual time courses very closely to the actual AVH time course.

Our results showed differences between hallucination and rest periods, indicating more erratic cortical behavior during AVH, as switching frequency increased and mean dwell time decreased during the hallucination periods. Moreover, differences in switch probability between AVH and rest periods point towards aberrant activation of the PL patterns. The combination of findings further provides support for the theory that AVH in clinical and non-clinical populations do not differ strongly regarding the mechanisms underlying brain activity during AVH on a group level. We were not able to distinguish the two groups based on most dynamic functional connectivity characteristics described. However, their dwell time did differ, which may be related to dysfunction of the pallidum in the clinical group. Altogether, this work offers valuable insights into the dynamic aspects of AVH in clinical and non-clinical populations.

## Funding

This work is supported by a grant awarded to I.S. from ZonMw mental health (GGZ) (ZonMw, project code: 63631 001 0). JC was funded by the Portuguese Foundation for Science and Technology, Portugal (UIDB/50026/2020, UIDP/50026/2020 and CEECIND/03325/2017) and by ”la Caixa” Foundation (LCF/BQ/PR22/11920014).

## CRediT authorship contribution statement

**Theresa M. Marschall:** Conceptualization, Methodology, Formal analysis, Writing – original draft, Visualization. **Sanne Koops:** Conceptualization, Writing – review & editing, Supervision. **Sanne G. Brederoo:** Conceptualization, Writing – review & editing, Supervision. **Joana Cabral:** Methodology, Software, Writing – review & editing. **Branislava Ćurčić-Blake:** Conceptualization, Writing – review & editing, Supervision. **Iris E.C. Sommer:** Conceptualization, Writing – review & editing, Supervision, Funding acquisition.

## Declaration of Competing Interest

The authors declare that they have no known competing financial interests or personal relationships that could have appeared to influence the work reported in this paper.

## Data Availability

The meta-data supporting the findings of this study are available on request from the corresponding author. The raw data are not available due to privacy restrictions.
