# Supplementary material for: Time varying dynamics of hallucinations in clinical and non-clinical voice-hearers
Source: Neuroimage Clin. 2023 Feb 14;37:103351. doi: 10.1016/j.nicl.2023.103351 (PMC9969260; doi:10.1016/j.nicl.2023.103351)
Supplement: Supplementary data 1 [file mmc1.docx]

# Appendix:

This file includes additional material for the article “Time varying dynamics of hallucinations in clinical and non-clinical voice hearers” by Marschall et al.

## 1.Rounding of the time courses:

We tested several rounding approaches implemented in matlab (round, ceil, floor, and a combination of the two latter) to find an array describing the time course of the individual AVH without losing much information. Round automatically rounds to the closest integer, whereas ceil rounds to the higher integer and floor to the lower.

### 1.1 Difference in duration

As seen in Figure A1 the difference between the original durations and the rounded durations were close to zero when using the round function implemented in matlab. A one sample t-test showed that the difference between the rounded and original durations did not differ significantly from 0 when using this approach (t(876) = -0.421, p =.674).


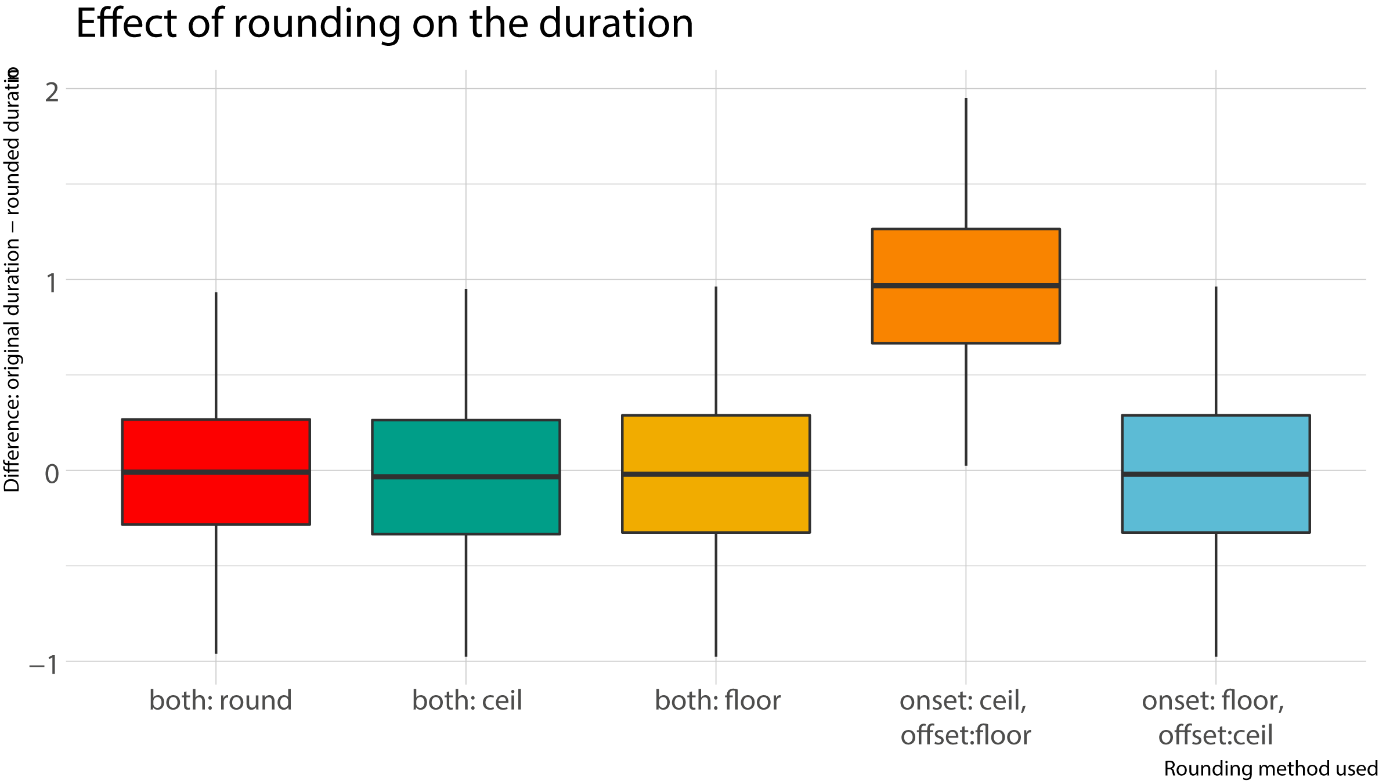


Figure A1: The effect of different rounding mechanisms on the duration. The x-axis depicts the different rounding methods. The y-axis the difference between the original duration and the rounded duration, this was calculated by subtracting the rounded duration from the original duration. The difference is measured in number of slices.

### 1.2 Difference in onset

As seen in Figure A2 the difference between the original onsets and the rounded onsets was the closest to zero when using the round function implemented in matlab. A one sample t-test showed that the difference between the rounded and original durations did not differ significantly from 0 when using this approach (t(876) = -0.623, p =.534).


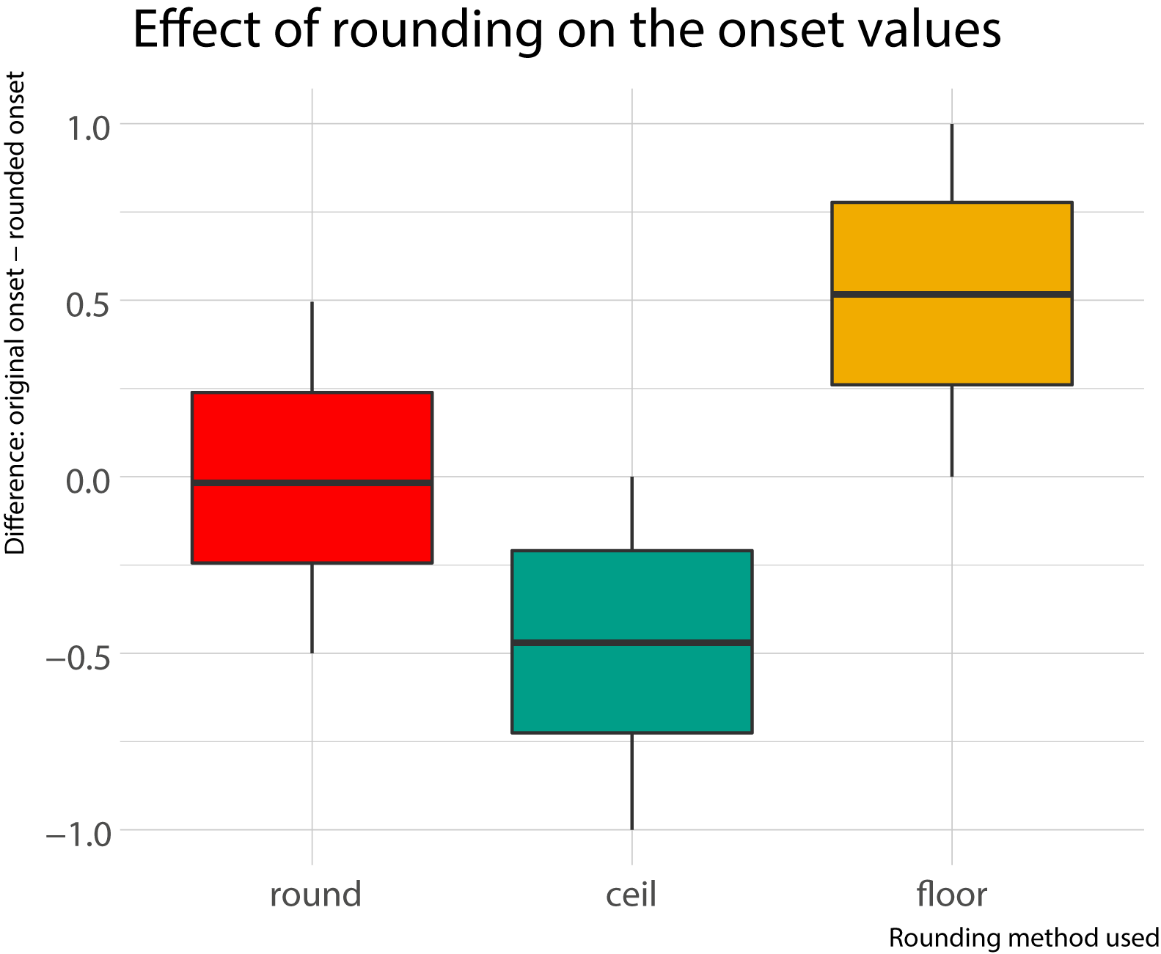


Figure A2: The effect of different rounding mechanisms on the onsets. The x-axis depicts the different rounding methods. The y-axis the difference between the original onsets and the rounded onsets, this was calculated by subtracting the rounded onsets from the original onsets. The difference is measured in number of slices.

### 1.3 Difference in offset

As seen in Figure A3 the difference between the original offsets and the rounded offsets was the closest to zero when using the round function implemented in matlab. A one sample t-test showed that the difference between the rounded and original durations did not differ significantly from 0 when using this approach (t(876) = -1.224, p =.221).


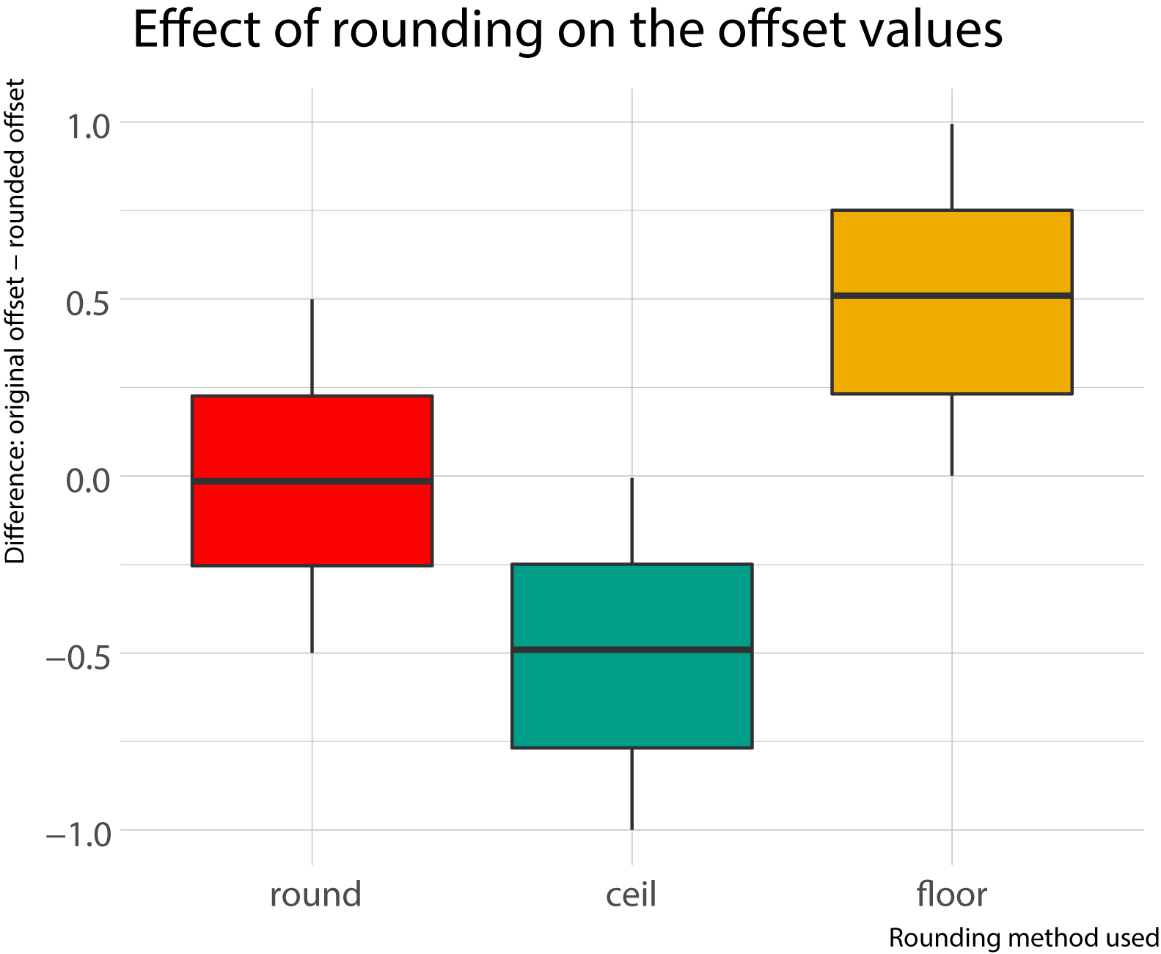


Figure A3: The effect of different rounding mechanisms on the offsets. The x-axis depicts the different rounding methods. The y-axis the difference between the original offsets and the rounded offsets, this was calculated by subtracting the rounded offsets from the original offsets. The difference is measured in number of slices.

## 2. Dunn index


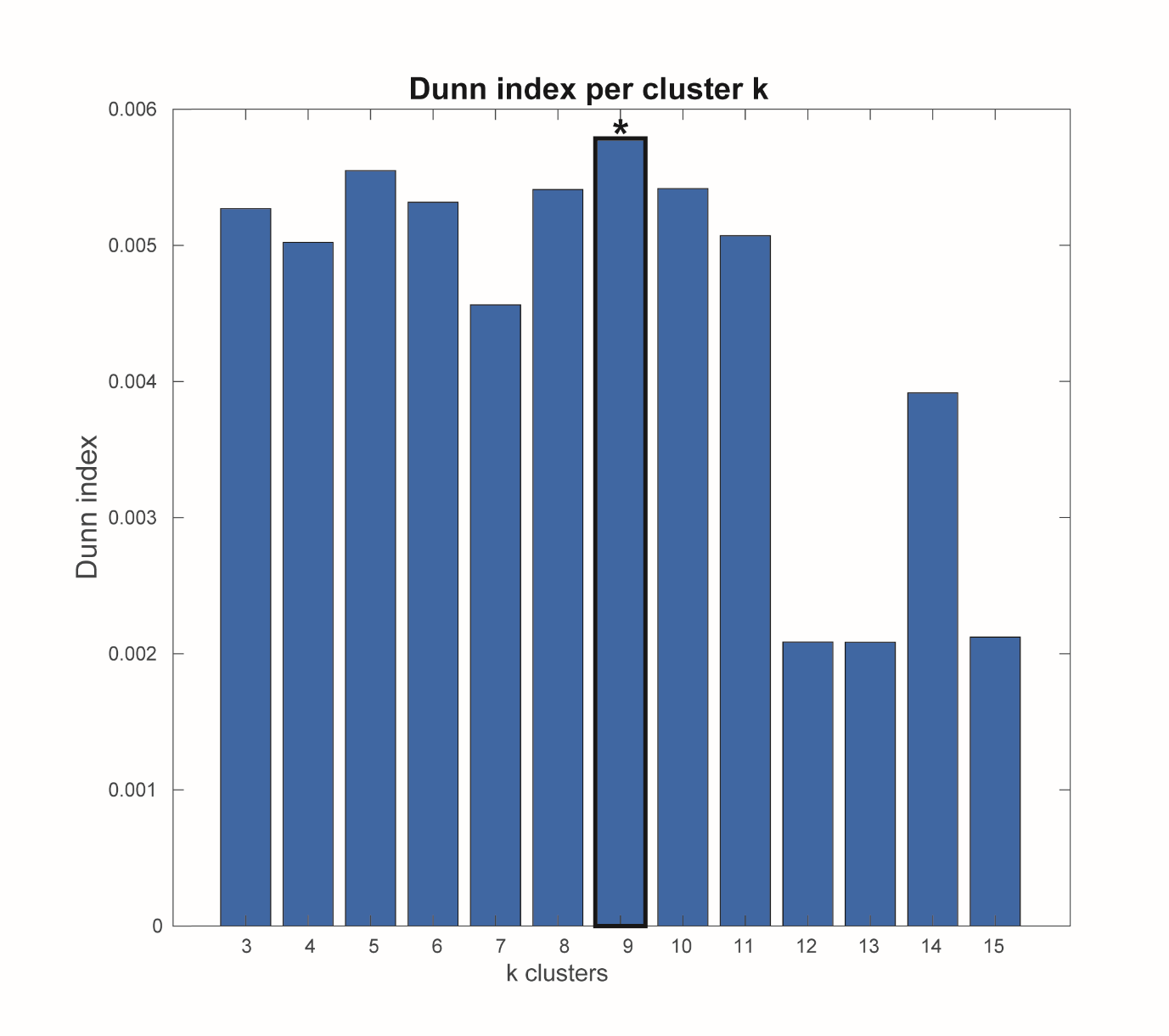


Figure A4: Dunn index of the different clusters considered for analysis. As k = 9 clusters (marked with asterisk) had the highest Dunn index this number of clusters was selected for further analysis. The x-axis shows the indexes for the different numbers of k considered. The y-axis shows the Dunn index

## 3. Positive eigenvalues of the leading eigenvectors for k = 9

Table A1: Positive eigenvalues of the leading eigenvectors with for each of the k = 9 PL. These eigenvalues are sorted in descending order and depict the phase locking patterns (PL) of interest. This allows to quickly identify areas most prominent in each of the PL. PL 8 is not included due to it consisting only of negative values. All values are also included in the excel file available as part of the appendix.

| **PL 1** | | | **PL 2** | | | **PL 3** | | | **PL 4** | | | **PL 5** | | | **PL 6** | | | **PL 7** | | | **PL 9** | | |
| --- | --- | --- | --- | --- | --- | --- | --- | --- | --- | --- | --- | --- | --- | --- | --- | --- | --- | --- | --- | --- | --- | --- | --- |
|  | **AAL label** | **V_1_** |  | **AAL label** | **V_1_** |  | **AAL label** | **V_1_** |  | **AAL label** | **V_1_** |  | **AAL label** | **V_1_** |  | **AAL label** | **V_1_** |  | **AAL label** | **V_1_** |  | **AAL label** | **V_1_** |
| R | Cuneus | 0.10 | L | Cuneus | 0.12 | R | Heschl | 0.12 | L | Front Mid Orb | 0.10 | L | Heschl | 0.09 | R | Pallidum | 0.08 | L | Fusiform | 0.11 | L | Front Inf Ope | 0.12 |
| R | Calcarine | 0.09 | L | Cingulum Post | 0.11 | L | Temporal Mid | 0.11 | R | Front Sup Med | 0.10 | L | Rolandic Oper | 0.08 | L | Olfactory | 0.07 | L | Front Inf Ope | 0.10 | L | Front Inf Tri | 0.11 |
| L | Cuneus | 0.09 | L | Precuneus | 0.11 | R | Rolandic Oper | 0.11 | R | Front Mid Orb | 0.09 | R | Rolandic Oper | 0.04 | L | Rectus | 0.06 | L | Occipital Inf | 0.10 | R | Supp Motor Ar | 0.11 |
| R | Lingual | 0.08 | L | Calcarine | 0.10 | R | SupraMarginal | 0.11 | L | Front Med Orb | 0.08 | R | Heschl | 0.04 | L | Cingulum Ant | 0.04 | L | Occipital Mid | 0.10 | L | Supp Motor Ar | 0.11 |
| R | Occipital Sup | 0.07 | L | Lingual | 0.10 | R | ParaHippocamp | 0.10 | L | Front Sup Orb | 0.08 | R | Insula | 0.04 | R | Rectus | 0.04 | L | Olfactory | 0.09 | R | Front Inf Tri | 0.10 |
| R | Precuneus | 0.07 | L | Cingulum Mid | 0.10 | R | Pallidum | 0.10 | R | Frontal Sup | 0.08 | L | Temporal Sup | 0.04 | L | Front Med Orb | 0.03 | L | Caudate | 0.09 | L | Paracentr Lob | 0.09 |
| L | Occipital Sup | 0.07 | R | Cingulum Ant | 0.09 | L | Amygdala | 0.09 | L | Frontal Sup | 0.08 | L | Insula | 0.03 | R | Olfactory | 0.03 | R | Occipital Inf | 0.09 | R | Frontal Sup | 0.09 |
| L | Calcarine | 0.06 | L | Thalamus | 0.09 | R | Fusiform | 0.09 | L | Rectus | 0.08 | L | Postcentral | 0.02 | R | Caudate | 0.01 | L | Angular | 0.08 | R | Front Inf Ope | 0.08 |
| R | ParaHippocamp | 0.04 | R | Front Sup Med | 0.09 | L | Cingulum Ant | 0.09 | L | Front Mid | 0.08 | R | Putamen | 0.01 | R | Cingulum Ant | 0.01 | L | Front Mid | 0.08 | R | Paracentr Lob | 0.08 |
| R | Cingulum Post | 0.04 | R | Paracentr Lob | 0.09 | L | SupraMarginal | 0.09 | R | Angular | 0.07 | R | SupraMarginal | 0.01 | R | Putamen | 0.00 | L | Temporal Mid | 0.08 | R | Front Mid | 0.07 |
| L | Lingual | 0.03 | R | Cingulum Mid | 0.08 | R | Occipital Mid | 0.08 | L | Front Sup Med | 0.07 | L | Front Inf Ope | 0.01 | L | Pallidum | 0.00 | L | Front Inf Tri | 0.08 | L | Insula | 0.06 |
| L | Cingulum Post | 0.01 | R | Supp Motor Ar | 0.08 | R | Postcentral | 0.08 | R | Front Med Orb | 0.07 | L | SupraMarginal | 0.00 |  |  |  | R | Occipital Mid | 0.08 | L | Frontal Sup | 0.04 |
| R | Thalamus | 0.00 | L | Paracentr Lob | 0.08 | R | Front Med Orb | 0.07 | L | Angular | 0.07 |  |  |  |  |  |  | R | Temporal Mid | 0.07 | R | Thalamus | 0.04 |
| R | Parietal Sup | 0.00 | R | Cuneus | 0.07 | R | Temporal Sup | 0.07 | L | Front Inf Orb | 0.07 |  |  |  |  |  |  | L | Front Med Orb | 0.06 | L | Front Mid | 0.03 |
|  |  |  | R | Front Med Orb | 0.07 | R | Cingulum Ant | 0.07 | R | Rectus | 0.06 |  |  |  |  |  |  | R | Putamen | 0.06 | L | Rolandic Oper | 0.03 |
|  |  |  | R | Thalamus | 0.07 | R | Temporal Mid | 0.06 | L | Front Inf Tri | 0.06 |  |  |  |  |  |  | L | Frontal Sup | 0.06 | L | Lingual | 0.02 |
|  |  |  | L | Occipital Sup | 0.06 | R | Tempr Pol Mid | 0.06 | R | Front Sup Orb | 0.04 |  |  |  |  |  |  | R | Amygdala | 0.06 | R | Front Sup Orb | 0.02 |
|  |  |  | R | Cingulum Post | 0.06 | R | Parietal Inf | 0.06 | R | Occipital Mid | 0.04 |  |  |  |  |  |  | L | Parietal Inf | 0.05 | L | Heschl | 0.01 |
|  |  |  | R | Precuneus | 0.06 | R | Angular | 0.06 | R | Front Mid | 0.02 |  |  |  |  |  |  | L | ParaHippocamp | 0.04 | R | Insula | 0.01 |
|  |  |  | L | Putamen | 0.05 | L | Putamen | 0.05 | R | Front Inf Orb | 0.02 |  |  |  |  |  |  | L | Precentral | 0.04 | L | ParaHippocamp | 0.00 |
|  |  |  | R | Caudate | 0.04 | L | Temporal Sup | 0.05 | R | Parietal Inf | 0.00 |  |  |  |  |  |  | L | Pallidum | 0.04 | L | Thalamus | 0.00 |
|  |  |  | R | Calcarine | 0.04 | R | Hippocampus | 0.05 |  |  |  |  |  |  |  |  |  | R | Fusiform | 0.04 |  |  |  |
|  |  |  | R | Frontal Sup | 0.03 | L | Postcentral | 0.05 |  |  |  |  |  |  |  |  |  | L | Front Sup Orb | 0.03 |  |  |  |
|  |  |  | R | Lingual | 0.02 | L | Occipital Inf | 0.05 |  |  |  |  |  |  |  |  |  | R | Temporal Sup | 0.03 |  |  |  |
|  |  |  | L | Supp Motor Ar | 0.01 | L | Pallidum | 0.05 |  |  |  |  |  |  |  |  |  | L | Rolandic Oper | 0.03 |  |  |  |
|  |  |  | L | Insula | 0.00 | L | Occipital Mid | 0.04 |  |  |  |  |  |  |  |  |  | L | Amygdala | 0.03 |  |  |  |
|  |  |  |  |  |  | R | Olfactory | 0.03 |  |  |  |  |  |  |  |  |  | L | Tempr Pol Sup | 0.03 |  |  |  |
|  |  |  |  |  |  | R | Occipital Sup | 0.03 |  |  |  |  |  |  |  |  |  | R | Temporal Inf | 0.01 |  |  |  |
|  |  |  |  |  |  | R | Amygdala | 0.03 |  |  |  |  |  |  |  |  |  | L | SupraMarginal | 0.01 |  |  |  |
|  |  |  |  |  |  | L | Precentral | 0.03 |  |  |  |  |  |  |  |  |  | L | Hippocampus | 0.00 |  |  |  |
|  |  |  |  |  |  | R | Precentral | 0.03 |  |  |  |  |  |  |  |  |  | L | Rectus | 0.00 |  |  |  |
|  |  |  |  |  |  | R | Caudate | 0.03 |  |  |  |  |  |  |  |  |  |  |  |  |  |  |  |
|  |  |  |  |  |  | L | Temporal Inf | 0.02 |  |  |  |  |  |  |  |  |  |  |  |  |  |  |  |
|  |  |  |  |  |  | R | Occipital Inf | 0.02 |  |  |  |  |  |  |  |  |  |  |  |  |  |  |  |
|  |  |  |  |  |  | L | Front Sup Med | 0.01 |  |  |  |  |  |  |  |  |  |  |  |  |  |  |  |

## 4. PL of the other k clusters and their correlation with the PL of k = 9


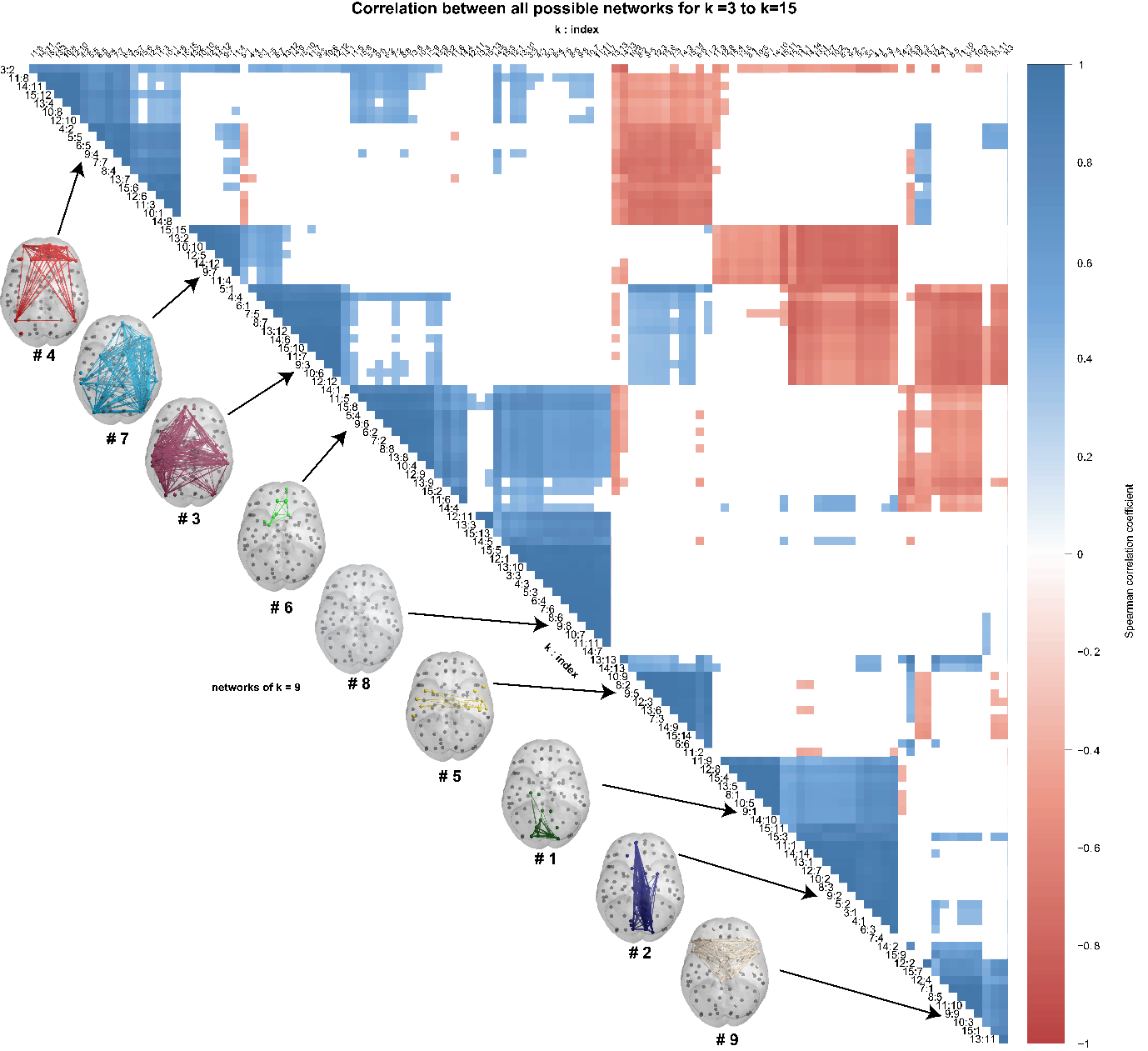


Figure A5: Correlation coefficients between all the clusters from the k = 3 to k = 15 cluster possibilities considered for analysis. Several PL correlate strongly and therefore similar PL were extracted for the different values for k. Only coefficients significant at p = .05/117 are shown, for the rest the cells are left white. Each row and column are labelled based on the number of k and the index within the k, e.g., the first PL from k=3 would be labelled “3:1”. The PL used for analysis, that is PL of k=9, are depicted, and arrows point towards their rows in the correlation matrix. Values are sorted based on the hierarchical clustering order as implemented in the corrplot package for R.

## 5. Alternative representation of PL


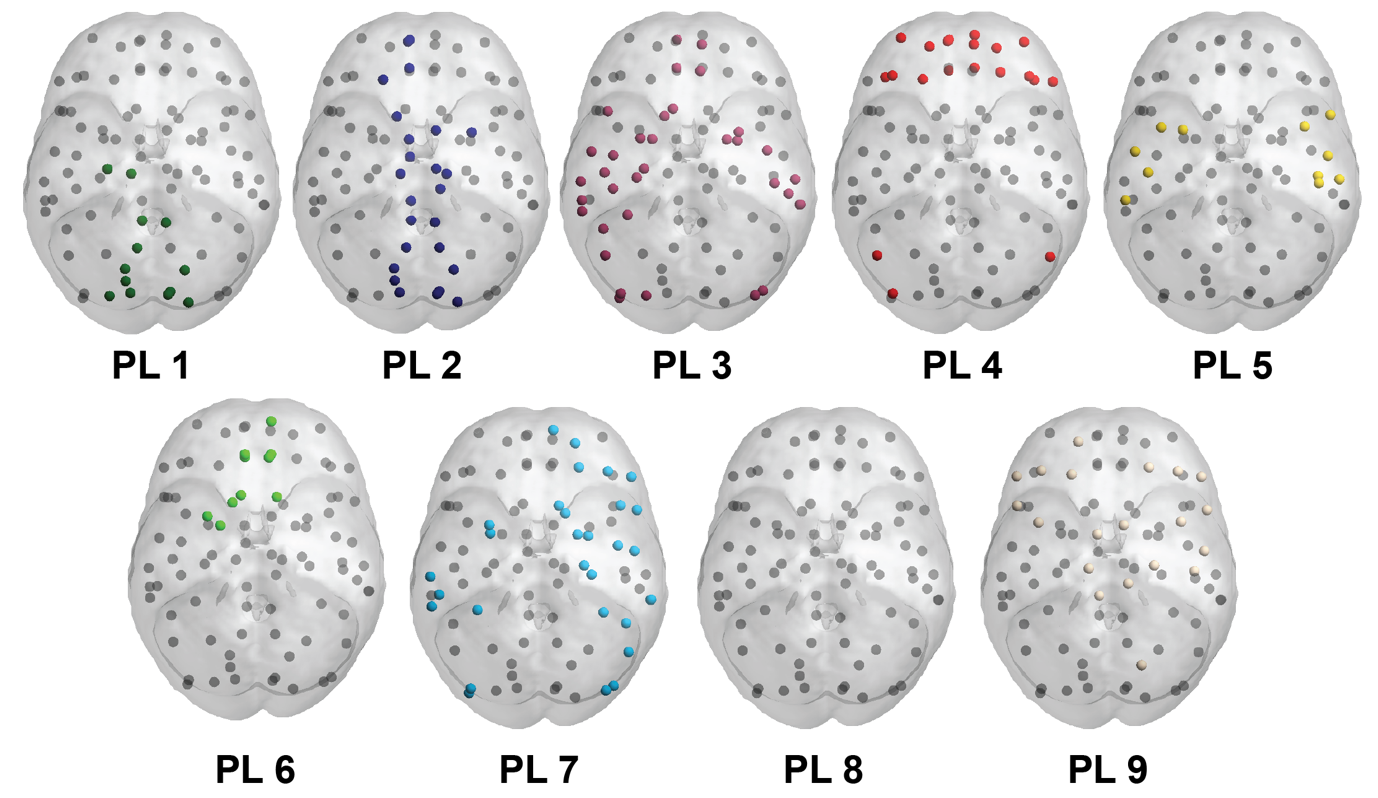


Figure A6: Alternative representation of the k = 9 PL states based on Figure 4 panel B of the main text. Graphical representation of the PLs in cortical space. Each sphere represents one of the ROIs of the AAL atlas. Colored spheres indicate the nodes of the PL.

## 6. Graphical representations of Yeo et al. atlas


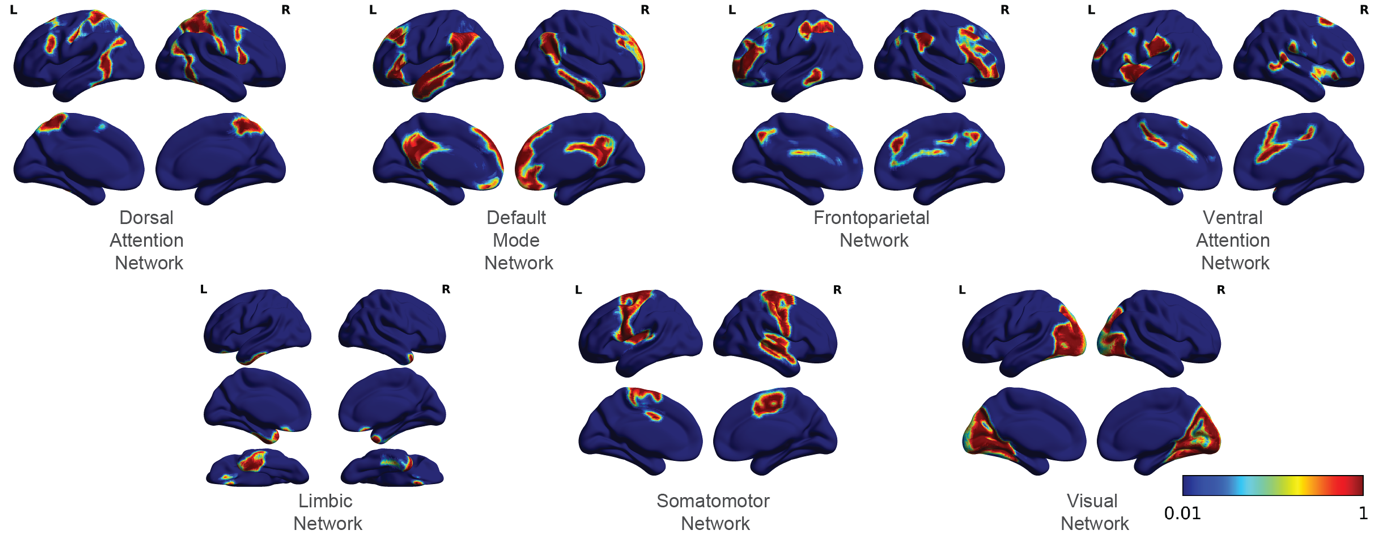


Figure A7: Overview of the 7 resting state networks as described by Yeo. et al. (2011) in the style of Figure 3 of the main text. Brain regions belonging to a given network are projected on an inflated cortex. An additional ventral view of the brain is included for the limbic network, to provide better visibility of the network.


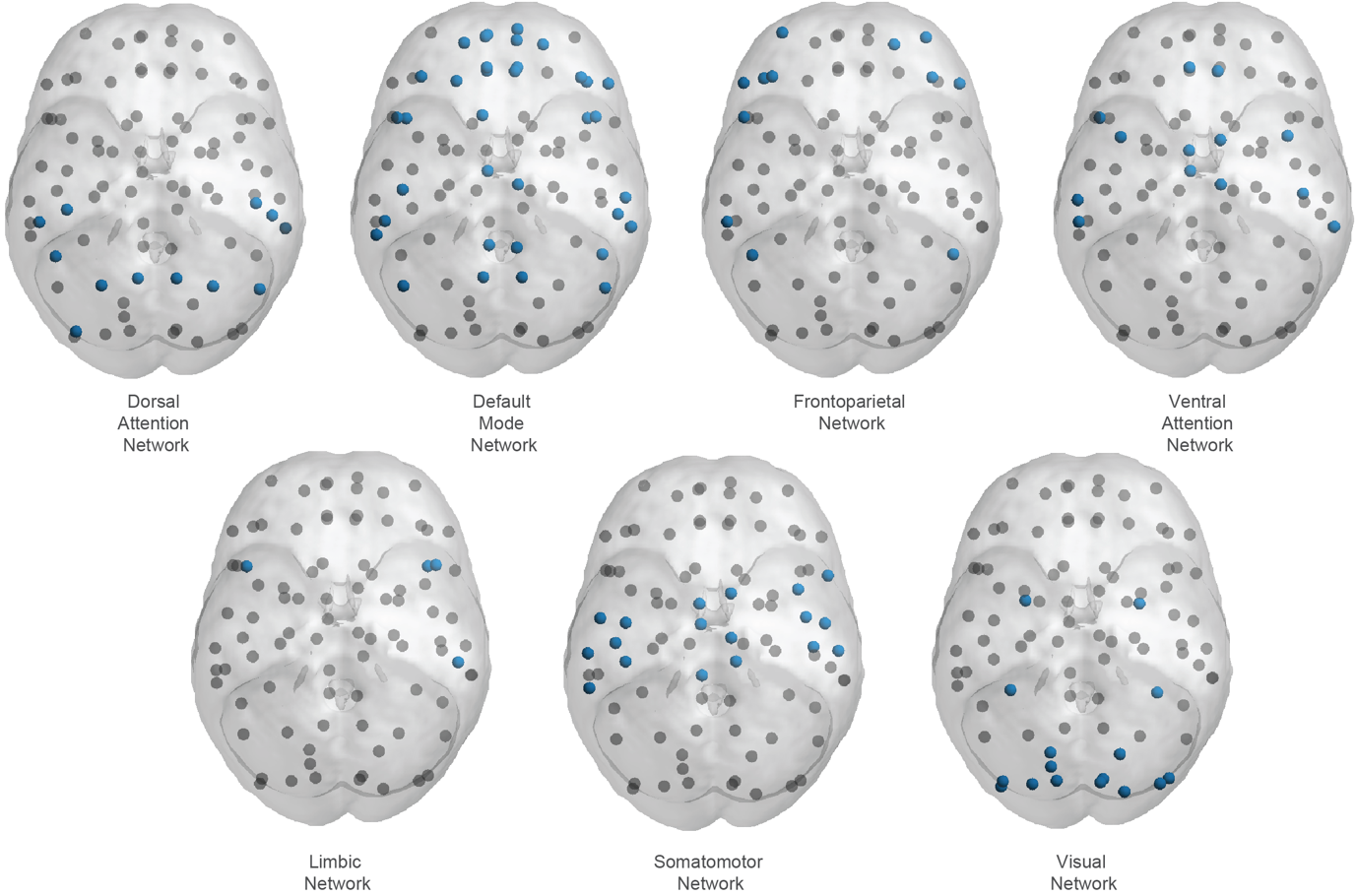


Figure A8: Overview of the 7 resting state networks as described by Yeo. et al. (2011) in the style of Figure 4 panel B of the main text. The networks were overlaid with the ROIs of the AAL atlas and representation in cortical space. At least 15% of voxels in the AAL ROIs marked in blue overlapped with the corresponding Yeo et al. (2011) network.

## 7. Distribution of cluster characteristics

### 7.1 Switching frequency:


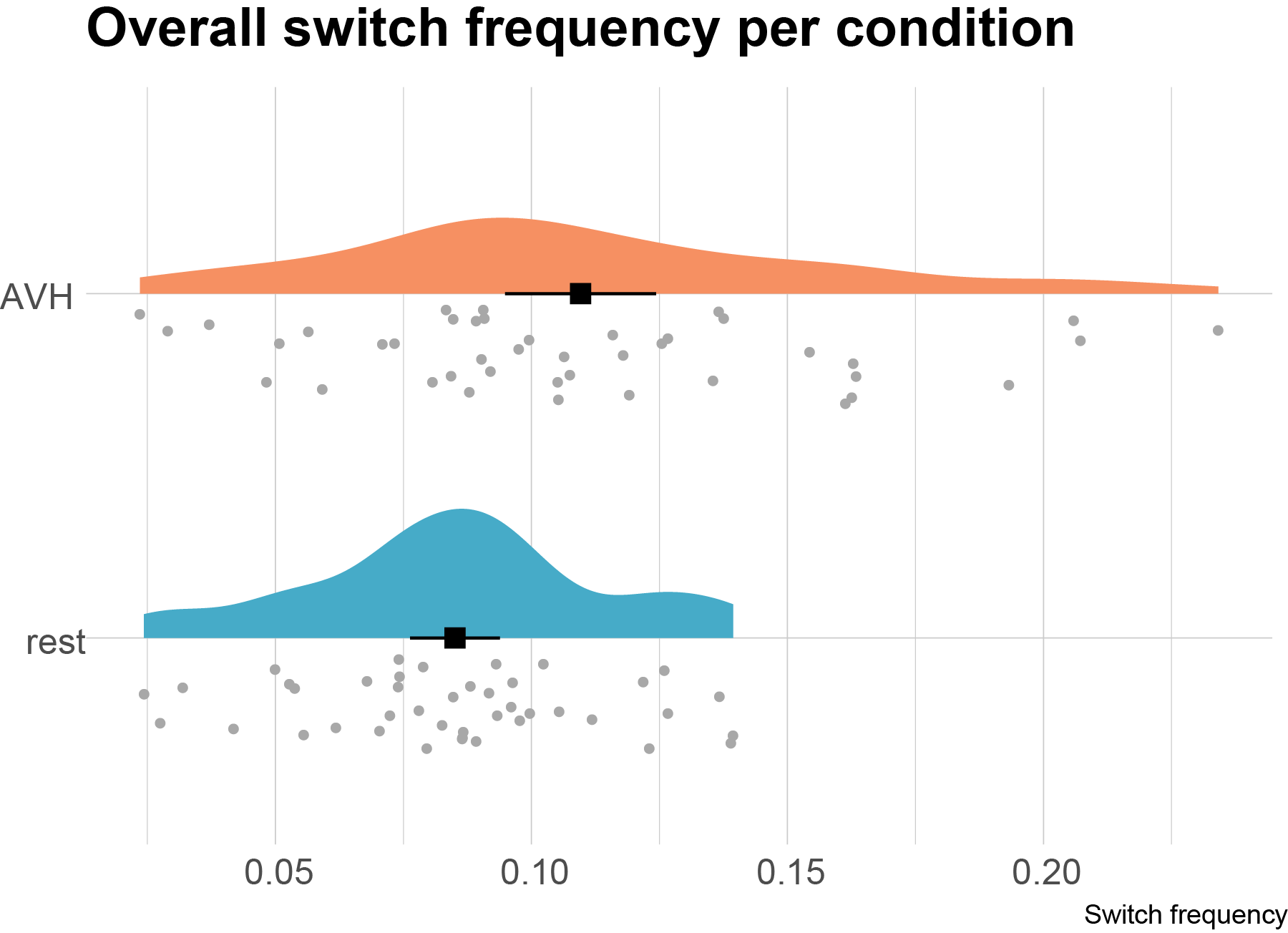


Figure A9: The switch frequency per condition. The y-axis shows the two conditions (AVH in orange, rest in blue). The x-axis depicts the switch frequency. Grey dots are the individual data points. Each data point corresponds to an occurrence of the AVH or rest per subject. Black boxes indicate the median of the distribution.


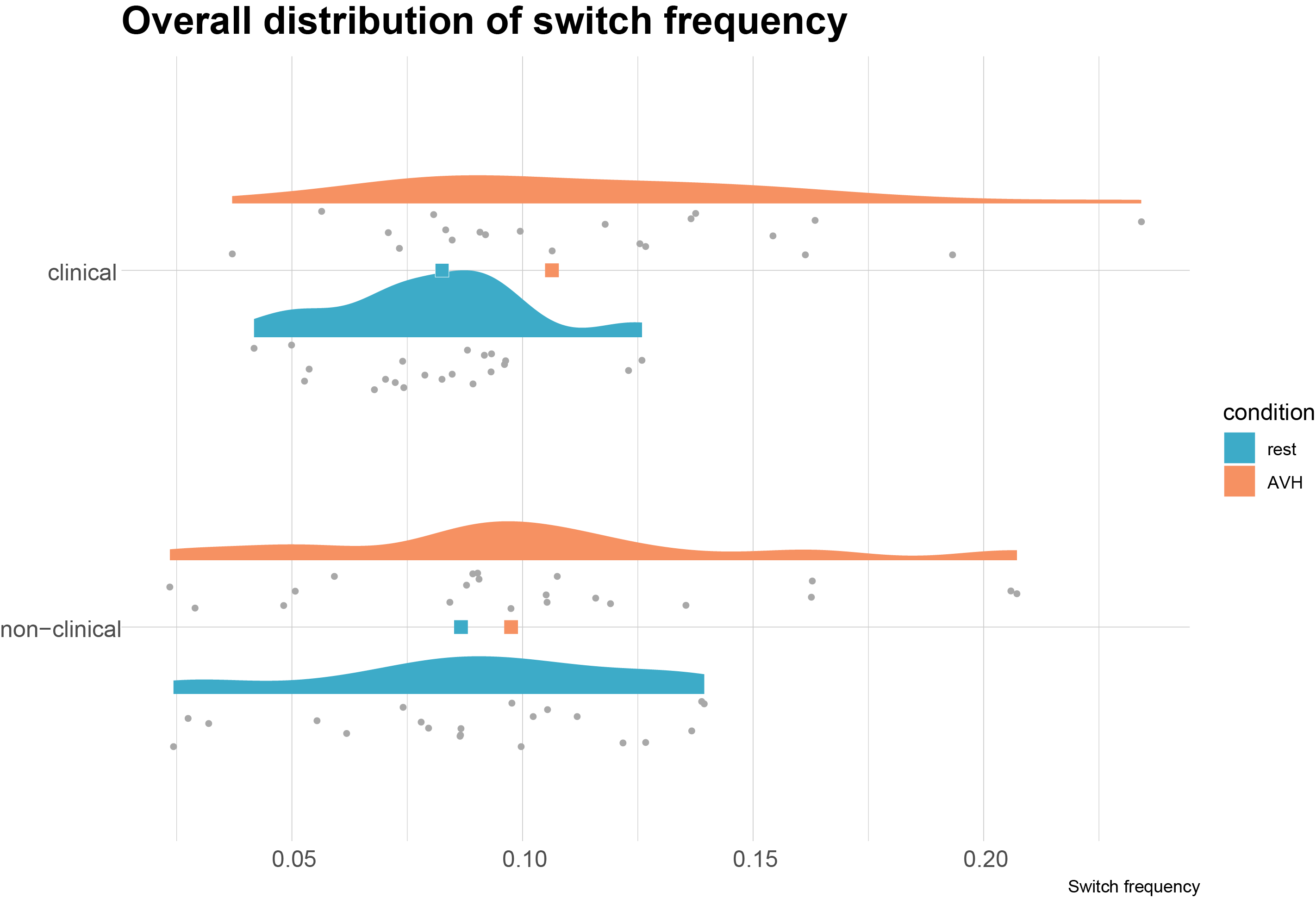


Figure A10: Violin plots and distribution of the individual values for the switch frequency. The x-axis shows the switch frequency. On the y-axis are the two groups, clinical and non-clinical voice hearers, of the sample. The color of the violin plot reflects the condition, with orange indicating AVH and blue the rest condition. The grey dots are the individual values. Medians per group per condition are depicted in between the violin plots.

### 7.2. Probability of occurrence:


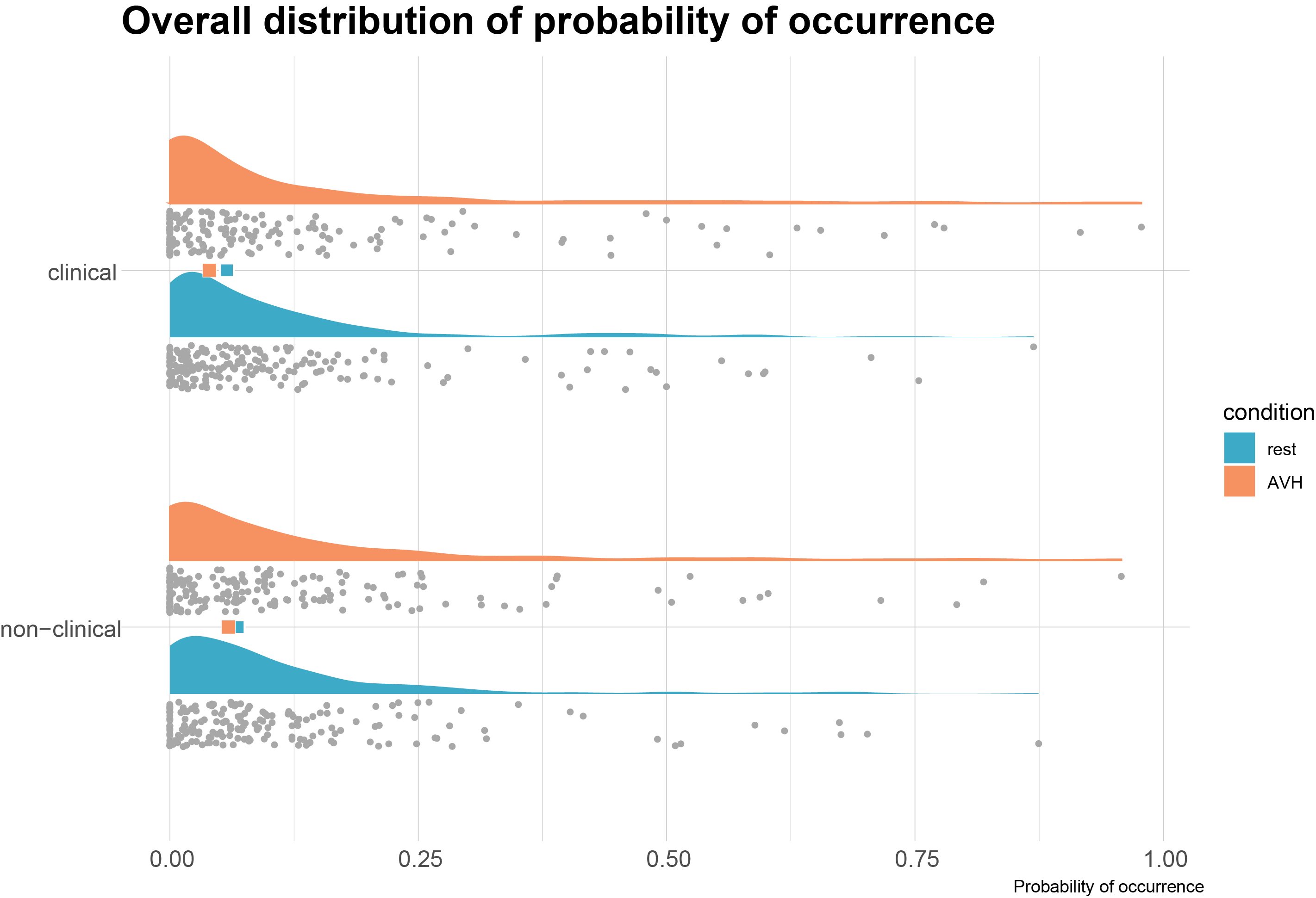


Figure A11: Violin plots and distribution of the individual values for the probability of occurrence. The x-axis shows the probability of occurrence. On the y-axis are the two groups, clinical and non-clinical voice hearers, of the sample. The color of the violin plot reflects the condition, with orange indicating AVH and blue the rest condition. The grey dots are the individual values. Medians per group per condition are depicted in between the violin plots.


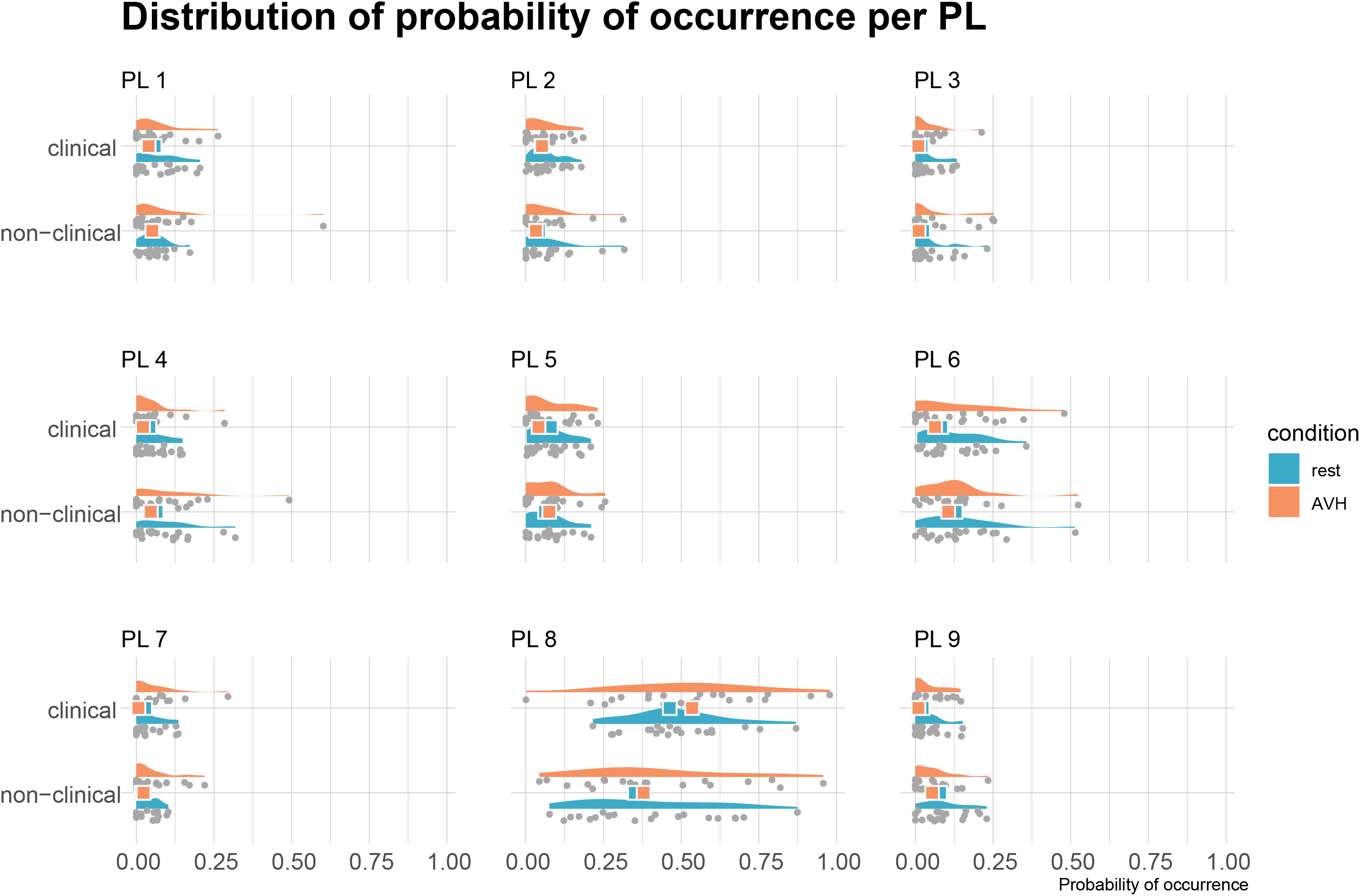


Figure A12: Violin plots and distribution of the individual values for the probability of occurrence per PL. Each panel depicts one of the PL. The x-axis per panel shows the probability of occurrence. On the y-axis are the two groups, clinical and non-clinical voice hearers, of the sample. The color of the violin plot reflects the condition, with orange indicating AVH and blue the rest condition. The grey dots are the individual values. Medians per group per condition are depicted in between the violin plots.

### 7.3. Mean dwell time:


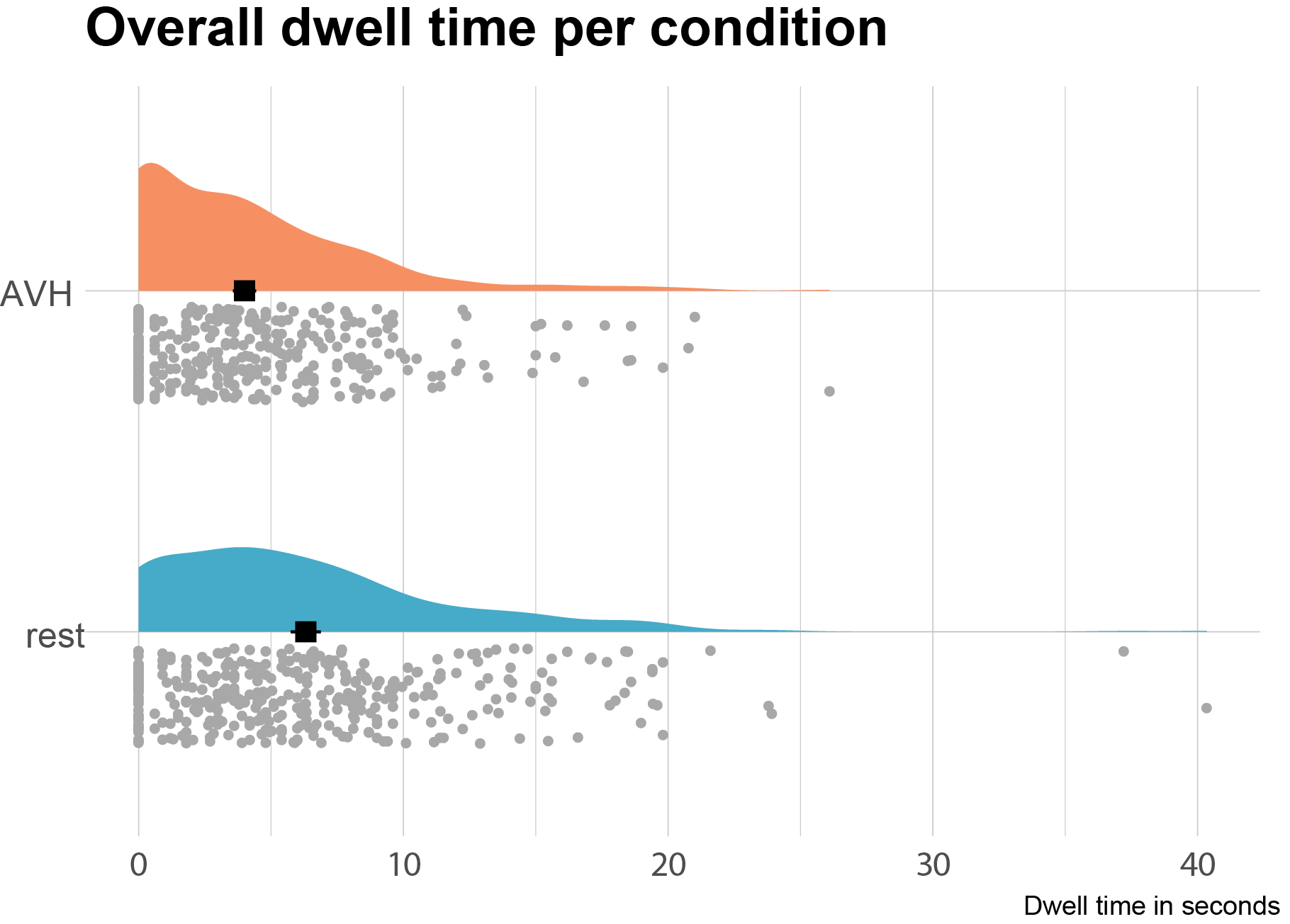


Figure A13: The dwell time per condition. The y-axis shows the two conditions (AVH in orange, rest in blue). The x-axis depicts the switch frequency. Grey dots are the individual data points. Switch frequency is measured as one value per subject per condition. Black boxes indicate the median of the distribution


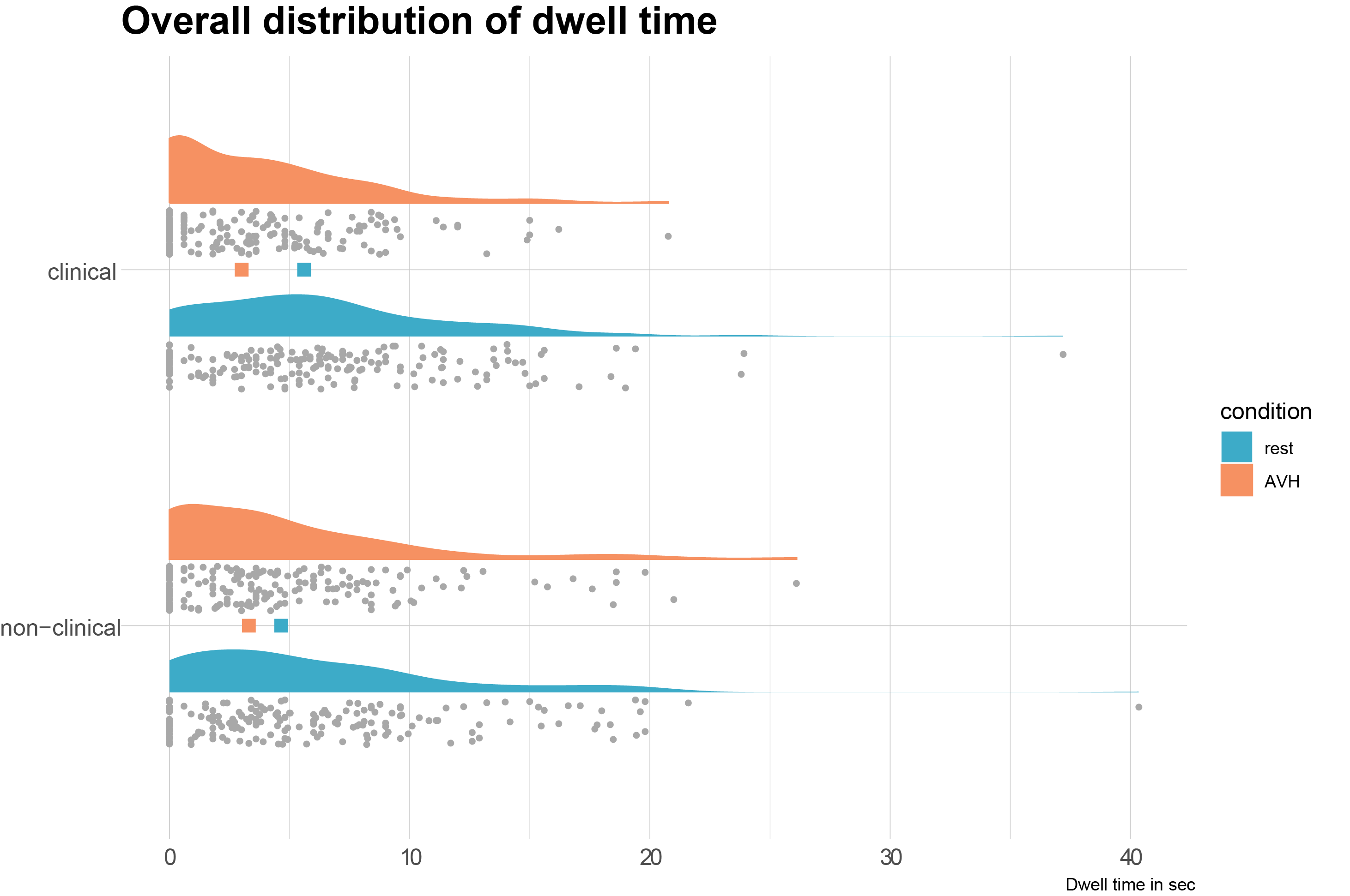


Figure A14: Violin plots and distribution of the individual values for the mean dwell time. The x-axis shows the mean dwell time. On the y-axis are the two groups, clinical and non-clinical voice hearers, of the sample. The color of the violin plot reflects the condition, with orange indicating AVH and blue the rest condition. The grey dots are the individual values. Medians per group per condition are depicted in between the violin plots.

### 7.4. Switching probability:


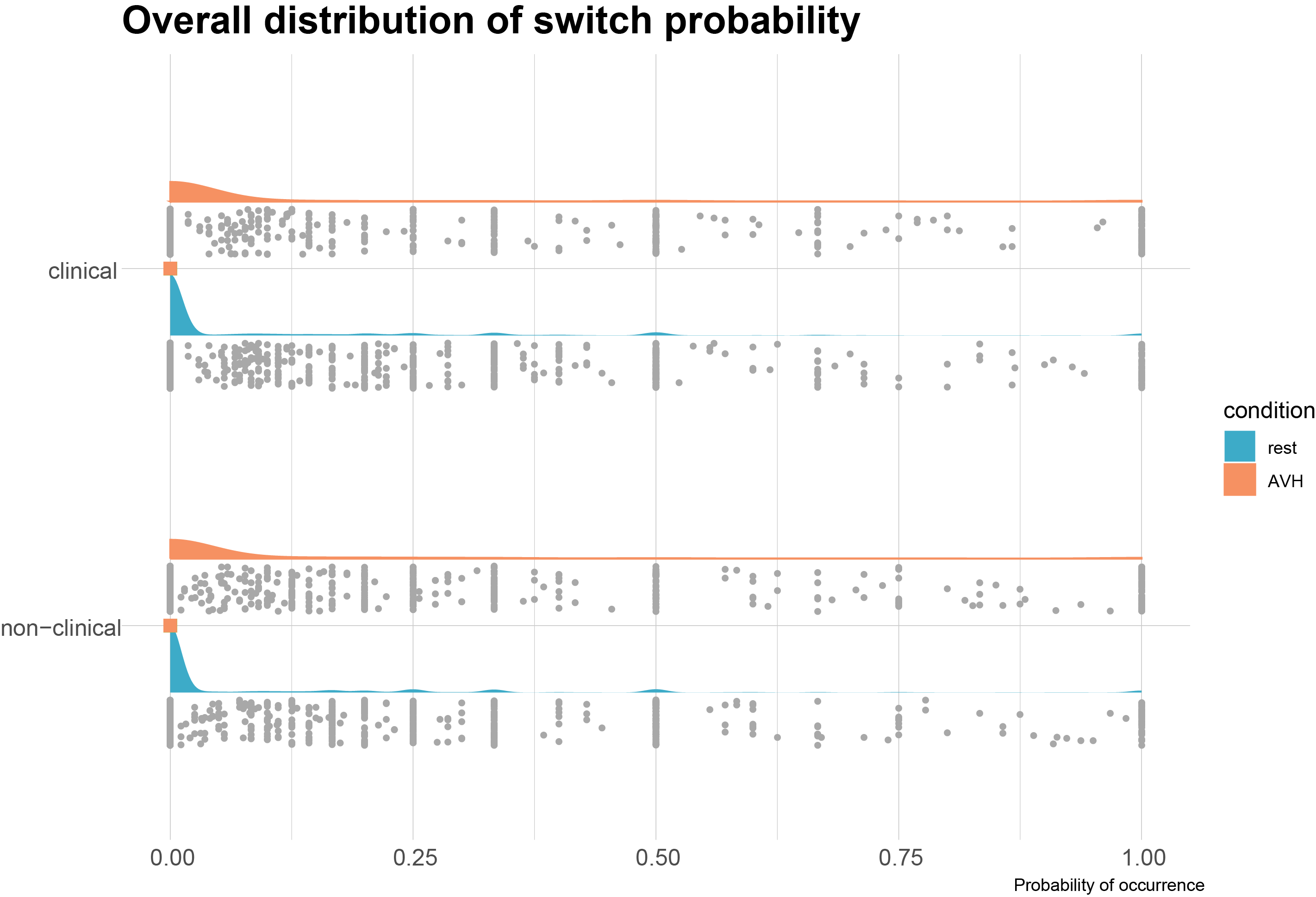


Figure A15: Violin plots and distribution of the individual values for the switch probability. The x-axis shows the switch probability. On the y-axis are the two groups, clinical and non-clinical voice hearers, of the sample. The color of the violin plot reflects the condition, with orange indicating AVH and blue the rest condition. The grey dots are the individual values. Medians per group per condition are depicted in between the violin plots.


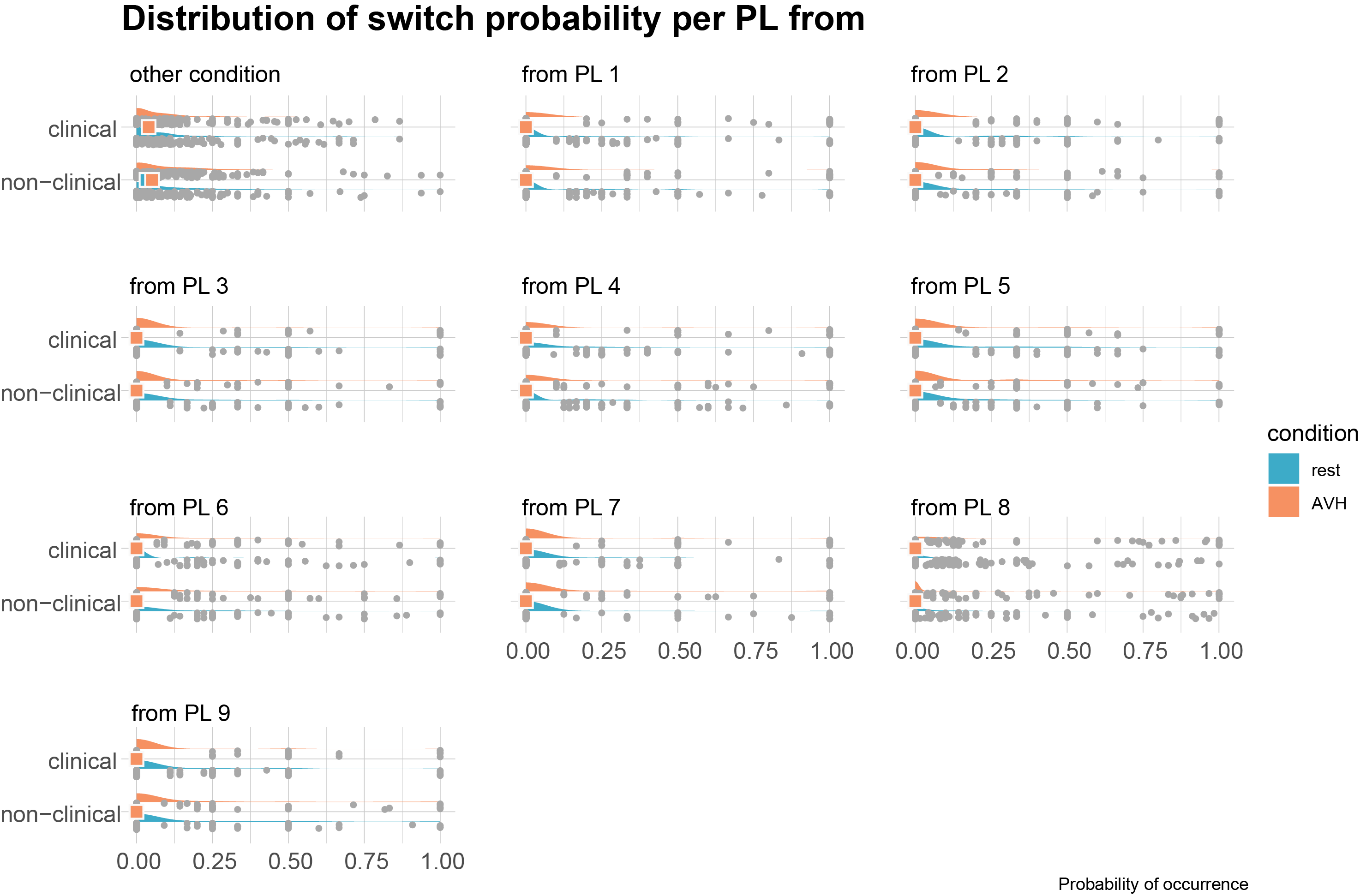


Figure A16: Violin plots and distribution of the individual values for the switch probability per PL the switch occurs from. Each panel depicts one of these PL. The x-axis per panel shows the switch probability. On the y-axis are the two groups, clinical and non-clinical voice hearers, of the sample. The color of the violin plot reflects the condition, with orange indicating AVH and blue the rest condition. The grey dots are the individual values. Medians per group per condition are depicted in between the violin plots.


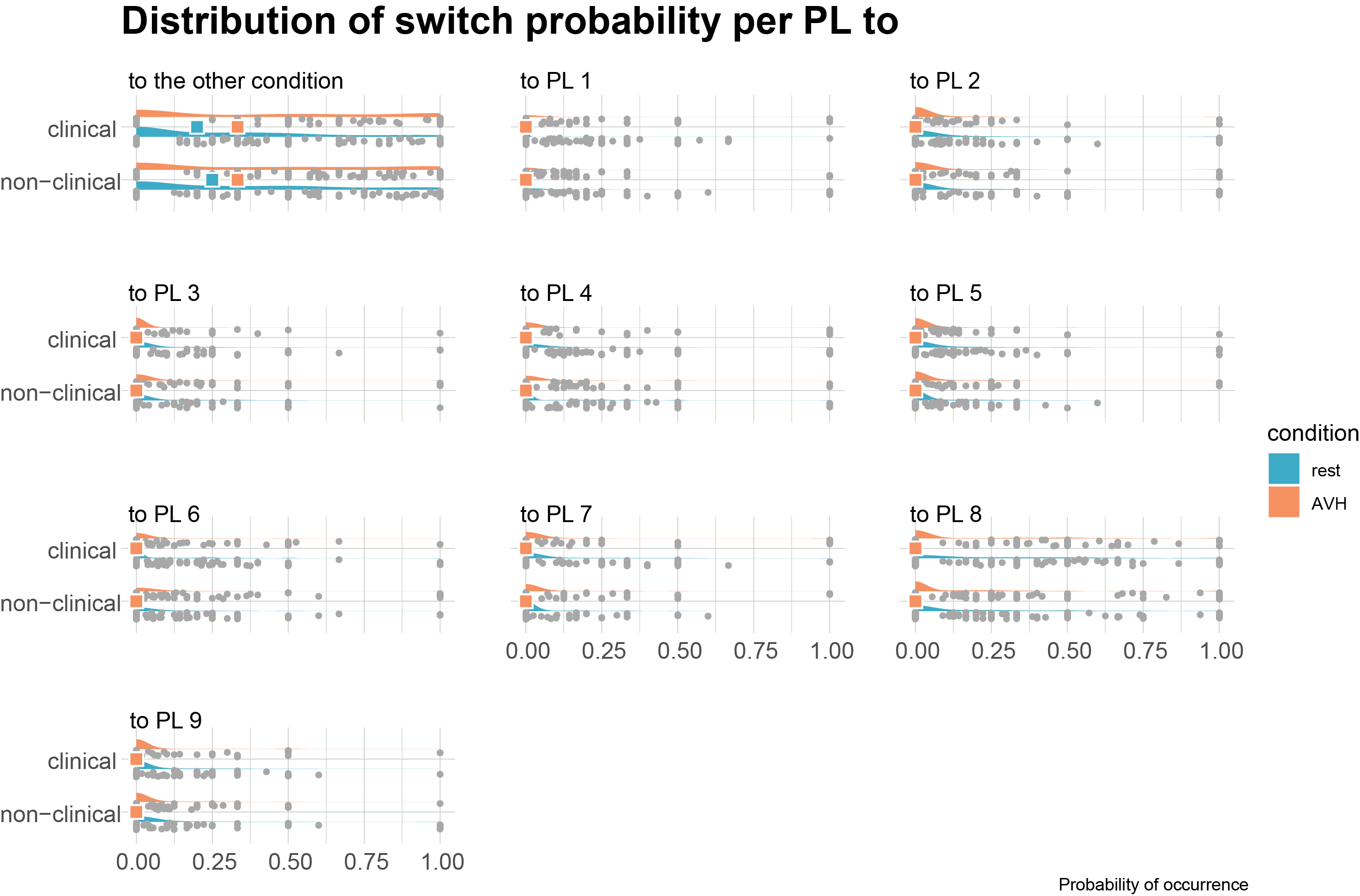


Figure A17: Violin plots and distribution of the individual values for the switch probability per PL the switch occurs to. Each panel depicts one of these PL. The x-axis per panel shows the switch probability. On the y-axis are the two groups, clinical and non-clinical voice hearers, of the sample. The color of the violin plot reflects the condition, with orange indicating AVH and blue the rest condition. The grey dots are the individual values. Medians per group per condition are depicted in between the violin plots.

## 8.Results ANOVA with shifted time course

## 8.1. Switching frequency:

The main effect of condition of the switching frequency was significant (F(1,40) = 12.576, p = .001), the switching frequency being higher during the occurrence of AVH (mean = .116) than during rest (mean = .085). Clinical and non-clinical voice-hearers did not differ from each other in terms of switching frequency (F(1,40) = .076, p = .781). The interaction between the two variables also showed no significant effect (F(1,40) = .625, p = .438).

## 8.2. Probability of occurrence:

There was no significant main effect of group (F(1,40) = .050, p = .716) or condition (F(1,40) = .447, p = .993) on probability of occurrence of clusters. Probability of occurrence showed a significant main effect of PL (F(8,320) = 69.750, p < .001), indicating that certain PLs were more likely to occur than others. PL 8 had the highest probability of occurrence with a mean of 45.65% and PL 3 the lowest with a mean of 4.38% followed by PL 7 with a mean of 4.52%. None of the interactions were significant (*group*condition*: F(1,40) = .013, p = .976; *group* PL*: F(8,320) = 1.779, p = .083; *condition* PL*: F(8,320) = 1.757, p = .085; *condition*group* PL*: F(8,320) = .933, p = .493).

## 8.3. Mean dwell time:

Mean dwell time in a cluster was higher during rest (mean = 6.332) than during AVH (mean = 3.881; F(1,40) = 24.232, p < .001). In addition, PL index significantly affected mean dwell time (F(8,320) = 15.022, p < .001). PL 8 had the highest dwell time with a mean of 9.651 and PL 3 the lowest with a mean of 3.273. There was no main effect of group (F(1,40) = .057, p =.809). There was a significant interaction between the three variables condition, group, and PL: (F(8,320) = 2.193, p = .025). None of the other interaction effects were significant (*group***condition*: F(1,40) = 1.232, p = .274; *group***PL*: F(8,320) = 1.282, p = .246; *condition***PL*: F(8,320) = 1.641, p = .110).

Further post hoc comparisons of the interactions between condition, group, and PL revealed a difference in the groups for PL 6 (p = . 002) and PL 8 (p = .002) during hallucinations. In both cases the clinical group (mean_PL6_ = 8.260; mean_PL8_ = 12.562) shows higher dwell times of the PL than the non-clinical group (mean_PL6_ = 2.939; mean_PL8_ = 7.201). The dwell time of PL 6 also differed between conditions for the clinical group (p = 0.03), with a higher dwell time during AVH (mean = 8.260) as compared to rest periods (mean = 6.946).

## 8.4. Switch probability:

There were no significant main effects of group (F(1,40) = .719, p = .329), condition (F(1,40) = .009, p = .920), the PL the switch occurred from (F(9,360) = .326, p = .879), or the PL the switch occurred to (F(9,360) = .098, p = .995) on the probability of transitioning from one state to another. Regarding the two-way interactions, there was only a significant effect of the PL the switch occurred from and the PL the switch occurred to (F(71,3240) = 9.631, p < .001), indicating that there are certain transition patterns that were more likely than others. The other two-way interactions were not significant (*group*condition*: F(1,40) = .282, p = .587; *group*PL_from*: F(9,360) = .789, p = .522; *group*PL_to*: F(9,360) = .298, p = .927; *condition*PL_from*: F(9,360) = .811, p = .514; *condition*PL_to*: F(9,360) = .673, p = .646). As for interactions including three variables we found an effect of group, *condition*, and *PL from* (F(9,360) = 3.414, p = .010), indicating that within one condition switches out of certain PL states were more common in one group over the other. The other interactions including three variables were not significant (*group*condition*PL_to*: F(9,360) = .567, p = .739; *group*PL_from*PL_to*: F(71,3240) = .707, p = .973; *condition*PL_from*PL_to*: F(71,3240) = 1.228, p = .096). Similarly, the interaction between all variables did not show a significant effect(F(71,3240) = .991, p = .501).

Further post hoc comparisons of the variables *group, condition*, and *PL to* revealed differences specifically regarding the switches out of the “other” condition compared to switches out of the actual Pls, however none of these differences survived FDR correction.
